# Perinuclear assembly of vimentin intermediate filaments induces cancer cell nuclear dysmorphia

**DOI:** 10.1016/j.jbc.2024.107981

**Published:** 2024-11-13

**Authors:** Ke-Wei Pan, Hong-Chen Chen

**Affiliations:** 1 Institute of Biochemistry and Molecular Biology, National Yang Ming Chiao Tung University, Taipei, Taiwan; 2 Cancer and Immunology Research Center, National Yang Ming Chiao Tung University, Taipei, Taiwan; 3 Department of Biotechnology and Bioindustry Sciences, National Cheng Kung University, Tainan, Taiwan

**Keywords:** vimentin, lamin, nuclear dysmorphia, EMT, DNA damage, genome instability

## Abstract

Nuclear dysmorphia, characterized by crumpled or lobulated polymorphic nuclear shapes, has been used as an index for the malignant grades of certain cancers. The expression of vimentin, a type-III intermediate filament protein, is a hallmark of the epithelial-to-mesenchymal transition. However, it remains unclear whether vimentin is involved in cancer cell nuclear dysmorphia. In this study, we found that vimentin intermediate filaments (VIFs) frequently accumulated at the concave of dysmorphic nucleus in breast cancer MDA-MB-231 cells. Depletion of vimentin apparently restored the nuclear shape of the cells, which was devastated by re-expression of vimentin, but not its assembly-defective Y117D mutant. Depletion of plectin, a cytoskeletal linker, partially prevented the perinuclear accumulation of VIFs and concomitantly restored the nuclear shape of the cells. In addition, depletion of vimentin in lung cancer A549 cells largely prevented nuclear dysmorphia during the epithelial-to-mesenchymal transition induced by TGFβ. Moreover, we found that VIF-mediated nuclear dysmorphia led to defects in DNA repair. Together, our results unveil a novel role of VIFs in cancer cell nuclear dysmorphia, which is associated with genome instability.

The mammalian cell nucleus usually exhibits as a spheroid or ellipsoid shape. However, some cancer cells display crumpled or lobulated polymorphic nuclear shapes, termed nuclear dysmorphia. Nuclear dysmorphia has been applied to clinical diagnosis for malignant grades of certain cancers (1). Since nuclear architecture is mainly supported by the nuclear lamina, a meshwork structure near the inner nuclear membrane, the rigidity of the nuclear lamina is thought to be critical for maintenance of nuclear shape (2). The nuclear lamina is composed of lamins (type V intermediate filament proteins) and nuclear membrane-associated proteins (3). Its integrity and rigidity can be affected by expression, post-translational modification, and mutation of lamins. Downregulation of lamin A has been shown to cause nuclear deformation in lung and breast cancer cells (4, 5). In most cases, phosphorylation of lamins at specific serine and tyrosine residues has an adverse effect on their assembly (6, 7, 8). The mutations in the *LMNA* gene cause a group of rare genetic disorders collectively called laminopathies (9). The cells derived from laminopathy patients display defects in the nuclear lamina organization and nuclear dysmorphia (10, 11).

Decreased rigidity of the nuclear lamina caused by defects in the nuclear lamina organization can render the nucleus susceptible to deformation by mechanical forces that directly apply to the nucleus. The forces that affect nuclear shape can arise from both outside and inside the nucleus. Outside the nucleus, the unrestrained actomyosin-mediated contractility was reported to drive cancer cell nuclear dysmorphia (12). Inside the nucleus, chromatin compaction *via* increased heterochromatin confers rigidity to the nucleus (13).

The defects in nuclear lamina organization can induce mislocalization and/or downregulation of DNA repair proteins, such as ATR and 53BP1, leading to genome instability (7, 14, 15, 16, 17). Therefore, nuclear dysmorphia is associated with defects in DNA repair and genome instability (18). Severe nuclear dysmorphia can even cause nuclear envelope rupture and micronuclei (12, 19). In addition, nuclear dysmorphia is one of the characteristic events during the epithelial-to-mesenchymal transition (EMT) (20), a process by which epithelial cells lose their cell polarity and cell-cell adhesion, and gain mesenchymal cell phenotypes, including enhanced migratory and invasive properties (21). Recently, nuclear dysmorphia was found to be concomitant with increased DNA damage and micronuclei during the EMT induced by transforming growth factor β (TGFβ) (7).

Vimentin, a type III intermediate filament protein, is mainly expressed in mesenchymal cells and its expression serves as a marker for EMT (22, 23). It was shown to be required for tumor progression and metastasis in a mouse model of non-small cell lung cancer (24). Vimentin intermediate filaments (VIFs) have been shown to play important roles in cell integrity, cell adhesion, cell motility, and invasion (25, 26, 27, 28, 29, 30). VIFs help to support cell motility *by* conferring viscoelastic characteristics to the cells during EMT (31). Besides, the VIF dynamics regulated by Src-mediated phosphorylation at Tyr117 is important for cell motility upon growth factor stimulation (32).

VIFs are indirectly linked to the nucleus through other cytoskeletal linkers and nuclear envelope proteins (33, 34). For example, plectin, a giant cytoskeletal linker that is associated and/or directly bound with the subcomponents of all three major cytoskeletons, has been reported to link VIFs to the nucleus (34, 35, 36). VIFs are involved in the formation of nuclear blebs and grooves in mouse embryonic fibroblasts (37). More recently, VIFs were found to regulate nuclear segmentation in neutrophils (38). However, it remains unexplored whether VIFs contribute to nuclear dysmorphia during EMT. Here, our study unveils the important role of VIFs in cancer cell nuclear dysmorphia and genome instability. We showed that the expression, assembly, and perinuclear accumulation of vimentin is necessary for it to induce nuclear dysmorphia and defects in DNA repair.

## Results

### Vimentin intermediate filaments often accumulate at the concave of dysmorphic nucleus in MDA-MB-231 cells

**Figure 1:**
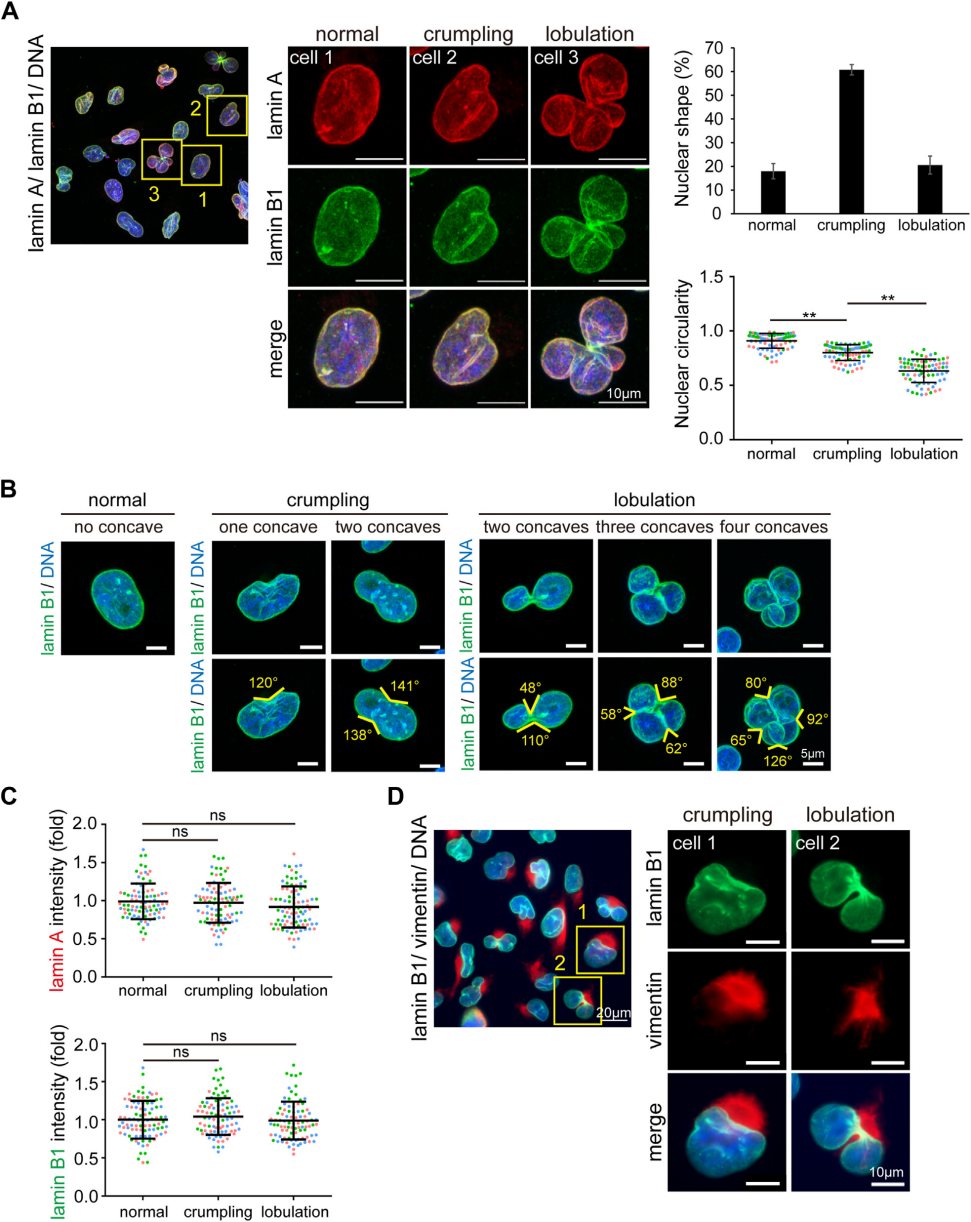
**Vimentin intermediate filaments frequently accumulate at the concave dysmorphic nucleus in MDA-MB-231 cells.***A*, MDA-MB-231 cells were stained for lamin A (*red*), lamin B1 (*green*), and DNA (*blue*). The immunofluorescence images were acquired using the Zeiss LSM880 confocal microscope imaging system. The insets respectively show normal, crumpled, and lobulated nuclei. Scale bars, 10 μm. The percentage of the cells with a normal, crumpled, or lobulated nucleus was measured (n ≥ 641). The nuclear circularity (4π x area/perimeter^2^) of the cells with a normal, crumpled, or lobulated nucleus was determined, respectively (n ≥ 100). *B*, MDA-MB-231 cells were stained for lamin B1 (*green*) and DNA (*blue*). The images were acquired using Zeiss LSM880 confocal microscope imaging system and the angles of the nuclear concaves were measured using the Zeiss ZEN2 software. Representative images are shown. Scale bars, 5 μm. *C*, the fluorescence intensities of lamin A and lamin B1 in normal, crumpled, and lobulated nuclei were measured (n ≥ 182) and expressed as –fold relative to the normal nucleus. *D*, MDA-MB-231 cells were stained for lamin B1 (*green*), vimentin (*red*), and DNA (*blue*). The images were acquired using Zeiss Apotome2 microscope imaging system. Representative images are shown. The insets show perinuclear accumulation of VIFs on crumpled and lobulated nuclei. Data information: In *A* and *C*, values (means ± SD) were from three independent experiments. ∗∗*p* < 0.01. ns, not significant.

The human breast cancer MDA-MB-231 cell line with a high ratio of nuclear dysmorphia was employed to study the mechanism of cancer cell nuclear dysmorphia. The nuclear shape was visualized by immunofluorescence staining for the nuclear lamina. Approximately 80% of MDA-MB-231 cells exhibited nuclear dysmorphia, characterized by crumpled (∼60%) or lobulated (∼20%) polymorphic nuclear shapes (Fig. 1*A*). Our criteria for a crumpled nucleus are: (i) a nucleus has only one concave with an angle ≦160° or (ii) a nucleus has two or more concaves with all angles between 90° and 160°. Instead, a nucleus that has two or more concaves with any of the angles ≦90° is defined as a lobulated nucleus (Fig. 1*B*). As expected, the nuclear circularity (4π x area/perimeter^2^) was inversely correlated with the severity of nuclear dysmorphia (Fig. 1*A*). The fluorescence intensities of lamin A and lamin B1 were not significantly different between normal and dysmorphic nuclei, suggesting that nuclear dysmorphia is less likely to result from aberrant expressions of lamin A and lamin B1 (Fig. 1, *A* and *C*). Interestingly, VIFs were frequently found to accumulate at the concave of the dysmorphic nucleus in MDA-MB-231 cells (Fig. 1*D*).

### The expression of vimentin is required and sufficient for nuclear dysmorphia in some cancer cell lines

**Figure 2:**
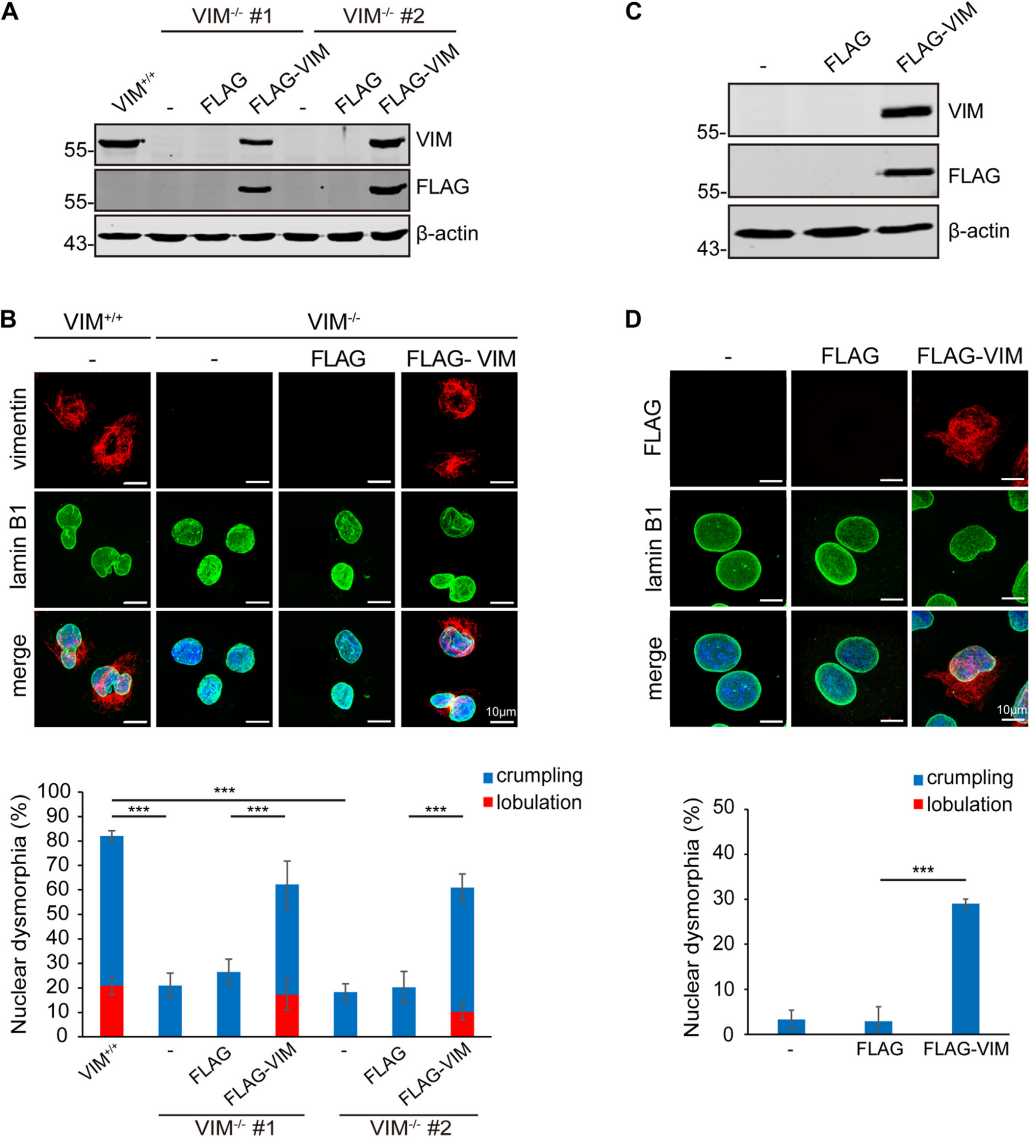
**The expression of vimentin is required and sufficient for nuclear dysmorphia in breast cancer cell lines.***A*, vimentin gene was edited in MDA-MB-231 cells using the CRISPR/Cas9 system. Two vimentin-deficient cell clones (VIM^−/−^ #1 and #2) were established and transiently transfected with the plasmid encoding FLAG-vimentin or the control vector for 24 h. An equal amount of whole cell lysates from parental MDA-MB-231 cells (VIM^+/+^) and the VIM^−/−^ cells was analyzed by immunoblotting with antibodies as indicated. *B*, the cells as described in (*A*) were stained for vimentin (*red*), lamin B1 (*green*), and DNA (*blue*). Representative images are shown. Scale bars, 10 μm. The percentage of crumpled or lobulated nucleus in MDA-MB-231 cells VIM^+/+^ and the VIM^−/−^ cells transiently expressing FLAG-vimentin was measured (n ≥ 231). *C*, MCF7 cells were transiently transfected with the plasmid encoding FLAG-vimentin or the control vector for 24 h. An equal amount of whole cell lysates was analyzed by immunoblotting with antibodies as indicated. *D*, The cells as described in (*C*) were stained for FLAG (*red*), lamin B1 (*green*), and DNA (*blue*). Representative images are shown. Scale bars, 10 μm. The percentage of crumpled or lobulated nucleus in MCF7 cells and those transiently expressing FLAG-vimentin was measured (n ≥ 208). Data information: In *B* and *D*, values (means ± SD) were from three independent experiments. ∗∗∗*p* < 0.001.

To examine the role of vimentin in nuclear dysmorphia, the vimentin gene was knocked out in MDA-MB-231 cells by the CRISPR/Cas9 system (Figs. 2*A* and S1). The depletion of vimentin apparently (∼75%) restored the nuclear shape of MDA-MB-231 cells, which was devastated by the reexpression of FLAG epitope-tagged vimentin (FLAG-vimentin) (Fig. 2*B*). To further confirm the role of vimentin in cancer cell nuclear dysmorphia, vimentin gene was knocked out in human pancreatic cancer PANC1 cells, ∼55% of which exhibited nuclear dysmorphia (Fig. S2). Similarly, vimentin gene knockout in PANC1 cells also significantly restored their nuclear shape (Fig. S2). Moreover, to examine whether vimentin is sufficient to induce nuclear dysmorphia, breast cancer MCF7 cells that do not express endogenous vimentin and exhibit normal nuclear shape were employed. The ectopic expression of FLAG-vimentin caused nuclear dysmorphia in ∼30% of the cells (Fig. 2, *C* and *D*). These results together indicate that vimentin is required and sufficient for nuclear dysmorphia at least in some cancer cells.

MDA-MB-231 cells retain the expression of keratins eight and 18. We found that depletion of keratins 8 or 18 by the small hairpin RNA (shRNA) approach did not affect the extent of nuclear dysmorphia in MDA-MD-231 cells (Fig. S3). Moreover, keratin 8 gene knockout by the CRISPR-Cas9 approach did not affect the extent of nuclear dysmorphia in MDA-MD-231 cells (Fig. S4). Therefore, the nuclear dysmorphia of MDA-MD-231 cells is not attributed to keratin intermediate filaments.

### The assembly of vimentin into filaments is necessary for it to induce nuclear dysmorphia

**Figure 3:**
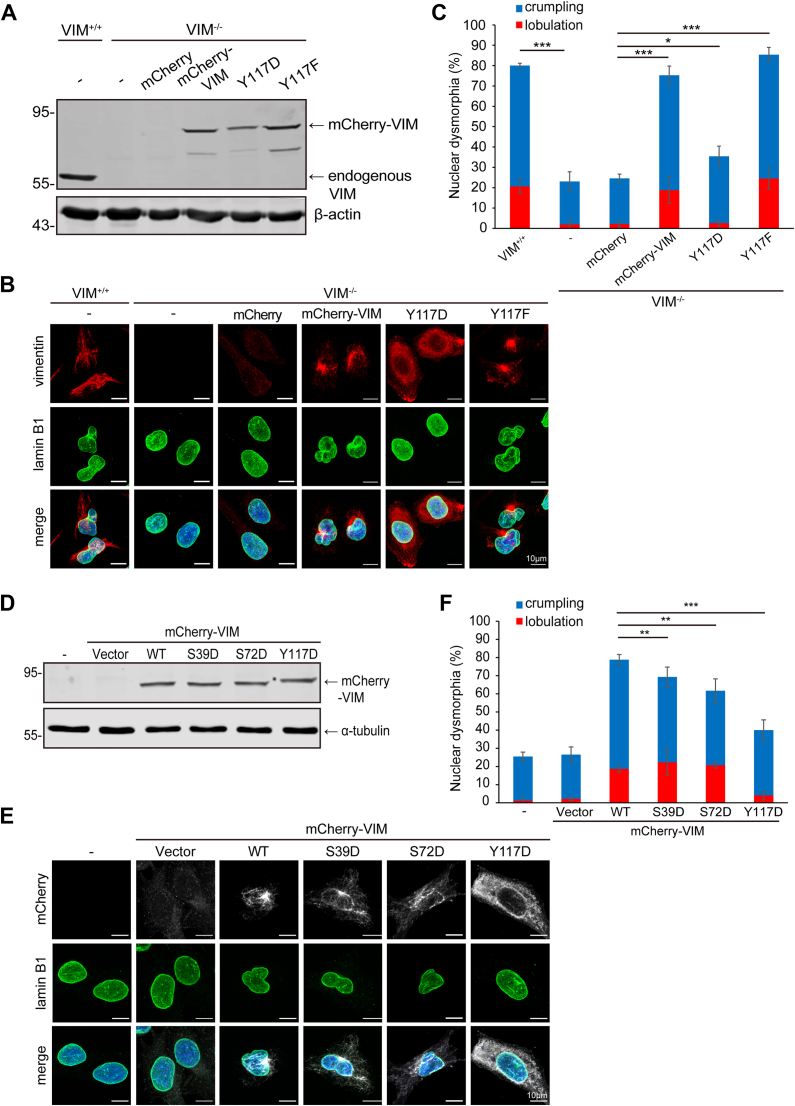
**The assembly of vimentin is necessary for nuclear dysmorphia in MDA-MB-231 cells.***A*, MDA-MB-231 VIM^−/−^ cells were infected by the lentiviruses expressing mCherry, mCherry-vimentin, or mCherry-vimentin Y117 mutants for 24 h and selected with neomycin (1 μg/ml) for 5 days. An equal amount of whole cell lysates from VIM^+/+^ cells, VIM^−/−^ cells, and those expressing mCherry or mCherry-vimentin was analyzed by immunoblotting with antibodies as indicated. *B* and *C*, The cells were stained with antibodies specific to vimentin (*red*), lamin B1 (*green*), and DAPI for DNA (*blue*). Representative images are shown. Scale bars, 10 μm. The percentage of crumpled or lobulated nucleus in the cells was measured (n ≥ 323). *D*, MDA-MB-231 VIM^−/−^ cells were transiently transfected with the plasmids encoding mCherry, mCherry-vimentin wild-type (WT), and the mutants (S39D, S72D, and Y117D) for 24 h. An equal amount of whole cell lysates was analyzed by immunoblotting with antibodies as indicated. *E* and *F*, The cells as described in (*D*) were stained with antibodies specific to vimentin (*white*), lamin B1 (*green*), and DAPI for DNA (*blue*). Representative images are shown. Scale bars, 10 μm. The percentage of dysmorphic nucleus in the MDA-MB-231 VIM^−/−^ cells transiently expressing mCherry or mCherry-vimentin was measured (n ≥ 216). Data information: In *C* and *F*, values (means ± SD) were from three independent experiments. ∗*p* < 0.05, ∗∗*p* < 0.01, ∗∗∗*p* < 0.001.

To examine whether the assembly of vimentin is necessary for it to induce nuclear dysmorphia, the assembly-deficient Y117D mutant of vimentin was employed. The Tyr117 of vimentin is known to serve as the phosphorylation site for Src kinase and the phosphomimetic Y117D mutant fails to assemble into filaments (32). The expression of mCherry fluorescent protein fused-vimentin (mCherry-vimentin) Y117D mutant into vimentin-depleted (VIM^−/−^) MDA-MB-231 cells mainly exhibited as particle-like structures and had little effect on the nuclear shape of the cells (Fig. 3, *A*–*C*), while both mCherry-vimentin WT and the Y117F mutant caused nuclear dysmorphia in MDA-MB-231 VIM^−/−^ cells, to the extent similar to that in their control counterpart cells (VIM^+/+^) (Fig. 3, *A*–*C*). Likewise, in MCF7 cells, the ectopic expression of mCherry-vimentin Y117D mutant had much less capability to induce nuclear dysmorphia than mCherry-vimentin WT and Y117F mutant did (Fig. S5). Moreover, it is known that the assembly of vimentin is affected by its phosphorylation at Ser39 and Ser72 (39, 40). Unlike the Y117D mutant, the phosphomimetic S39D and S72D mutants retained some capability to assemble into filaments (Fig. 3, *D* and *E*). Our results showed that the capability of mCherry-vimentin S39D and S72D mutant to induce nuclear dysmorphia in MDA-MB-231 VIM^−/−^ cells was slightly weaker than mCherry-vimentin WT, but much stronger than the Y117D mutant (Fig. 3, *E* and *F*). These results suggest that the assembly of vimentin is important for it to induce nuclear dysmorphia. It is worth noting that mCherry-vimentin also induced a morphological change of MCF7 cells from cuboidal to mesenchymal cell shape (Fig. S6), as described previously (26, 30).

### Plectin-mediated perinuclear accumulation of VIFs is important for nuclear dysmorphia in MDA-MB-231 cells

**Figure 4:**
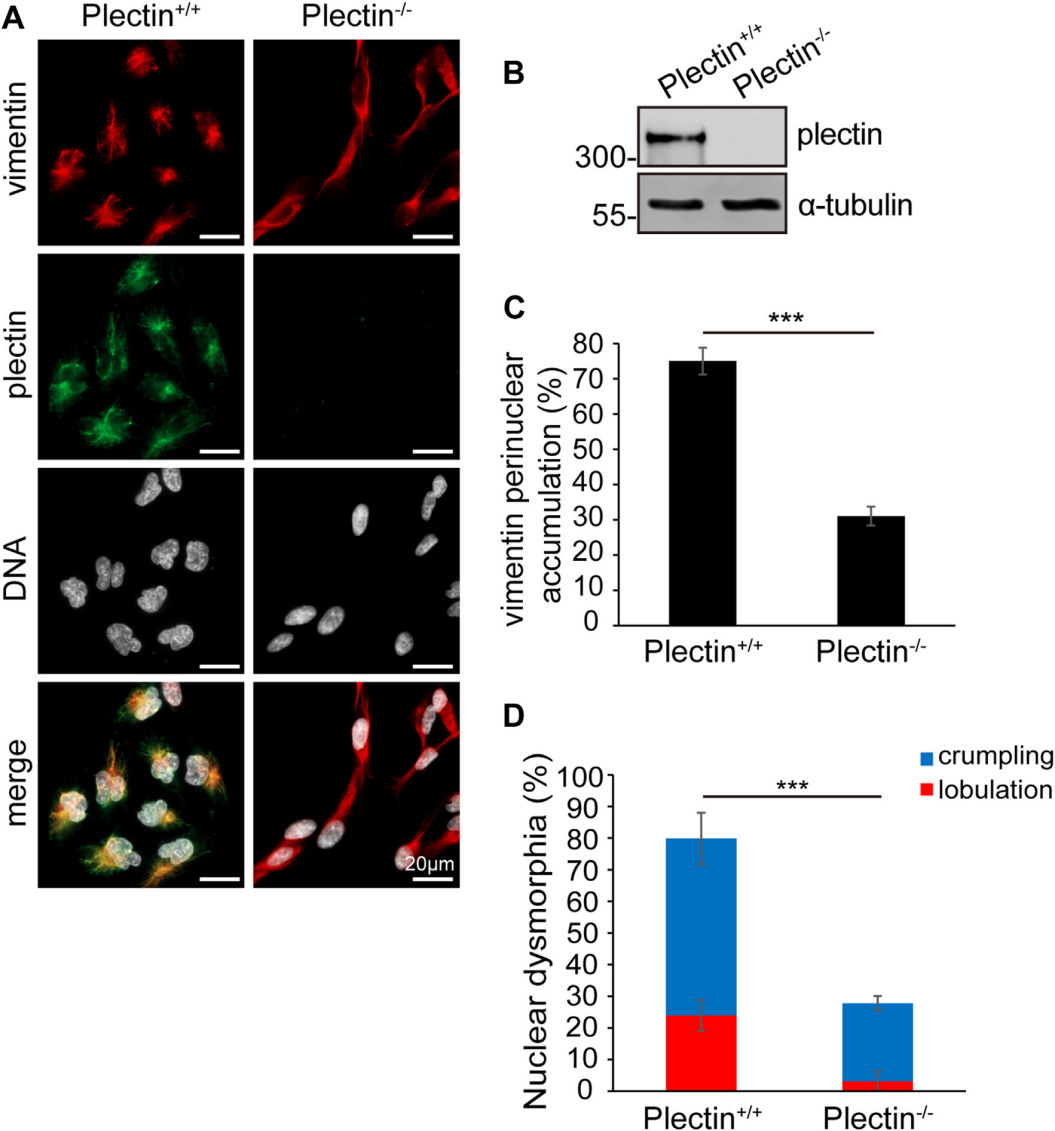
**The perinuclear accumulation of VIFs is important for nuclear dysmorphia in MDA-MB-231 cells.***A*, MDA-MB-231 cells deficient in plectin expression (plectin^−/−^) were established using the CRISPR/Cas9 system. MDA-MB-231 Plectin^+/+^ cells and Plectin^−/−^ cells were stained for vimentin (red), plectin (green) and DNA (blue). Scale bars, 20 μm. *B*, an equal amount of whole cell lysates was analyzed by immunoblotting with antibodies as indicated. *C*, the percentage of the cells with VIF perinuclear accumulation was measured (n ≥ 543). The distribution of VIFs within a 25 × 25 μm perinuclear area is defined as perinuclear accumulation. *D*, the percentage of the cells with a crumpled or lobulated nucleus was measured (n ≥ 389). Data information: In *C* and *D*, values (means ± SD) were from three independent experiments. ∗∗∗*p* < 0.001.

It is known that VIFs are indirectly linked to the nucleus through plectin (34, 35, 36). Indeed, we found that VIFs were largely colocalized with plectin at the concave of the dysmorphic nucleus in MDA-MB-231 cells (Fig. 4*A*), raising a possibility that the nuclear dysmorphia may result from compression by perinuclear VIFs. To examine this possibility, plectin was depleted by shRNAs specific to plectin in MDA-MB-231 cells (Fig. S7*A*). The shRNA-mediated depletion of plectin partially prevented the perinuclear accumulation of VIFs (Fig. S7, *B* and *C*) and restored ∼35% of the dysmorphic nuclei to their normal shape (Fig. S7*D*). To further confirm this, the plectin gene was knocked out by the CRISPR/Cas9 system in MDA-MB-231 cells (Figs. 4, *A* and *B*; S8). Compared to the shRNA-mediated approach, the depletion of plectin by the gene knockout approach was more apparently prevented the perinuclear accumulation of VIFs and restored the nuclear shape of MDA-MB-231 cells (Fig. 4, *C* and *D*). These results suggest that plectin-mediated perinuclear accumulation of VIFs may cause cancer cell nuclear dysmorphia.

### F-actin, microtubule, or heterochromatin is not involved in nuclear dysmorphia of MDA-MB-231 cells

Our experiments to this point demonstrate that the perinuclear accumulation of VIFs is important for nuclear dysmorphia in MDA-MB-231 cells, but it is not clear whether other factors are also involved in this regard. The unrestrained actomyosin-mediated contractility was reported to induce nuclear dysmorphia (12). However, the treatment of MDA-MB-231 cells with the F-actin depolymerization agent cytochalasin D, the myosin ATPase inhibitor blebbistatin, the Rho-associated kinase (ROCK) inhibitor Y-27632, or the myosin light-chain kinase (MLCK) inhibitor ML-7 did not restore their nuclear shape (Fig. S9*A*). In addition, microtubule was also reported to control nuclear shape (41), yet the treatment of MDA-MB-231 cells with the microtubule polymerization inhibitor nocodazole did not have any effect on their nuclear shape (Fig. S9*B*). Furthermore, the content of heterochromatin and euchromatin has been shown to be involved in nuclear deformation (13). To measure the content of heterochromatin, an antibody specific to the tri-methylation of lysine 27 on histone H3 protein (H3K27me3), a facultative heterochromatin marker (42), was used for immunofluorescence staining. As expected, the treatment of MDA-MB-231 cells with the broad histone methyltransferase inhibitor 3-Deazaneplanocin-A (DZNep) decreased their content of heterochromatin but did not affect the ratio of the nuclear dysmorphia of MDA-MB-231 cells (Fig. S9*C*). These results together suggest that the nuclear dysmorphia of MDA-MB-231 cells is less likely caused by mechanical forces generated by the F-actin and microtubule cytoskeleton or facultative heterochromatin.

### VIFs are involved in nuclear dysmorphia during EMT

**Figure 5:**
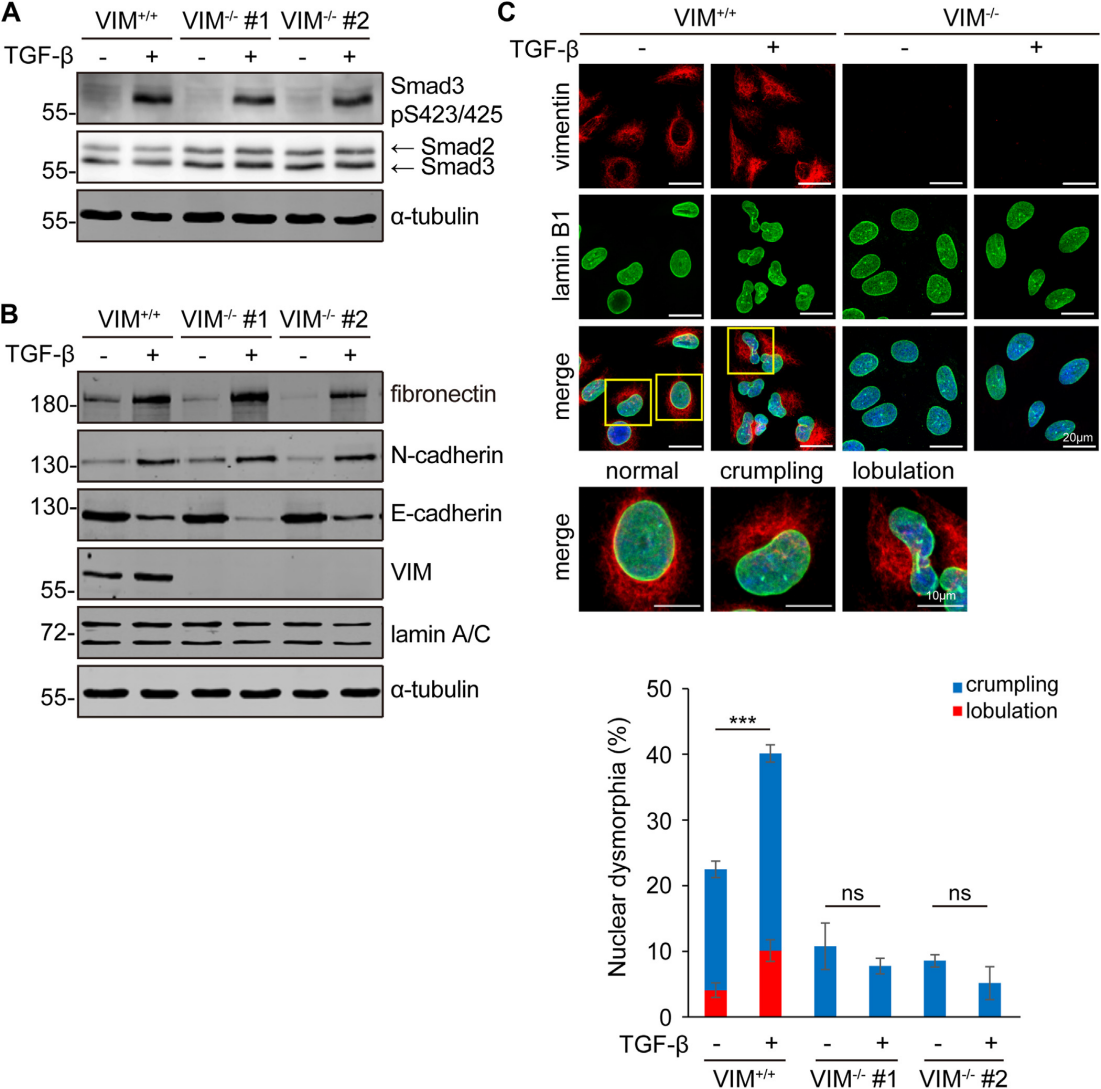
**VIFs are necessary for nuclear dysmorphia during TGFβ-induced EMT.***A* and *B*, Vimentin gene was edited in A549 cells using the CRISPR/Cas9 system. Two vimentin-deficient cell clones (VIM^−/−^ #1 and #2) were established. A549 VIM^+/+^ and VIM^−/−^ cells were treated with (+) or without (−) TGFβ (5 ng/ml) for 1 h (*A*) or 48 h (*B*) and then lyzed. An equal amount of whole cell lysates was analyzed by the immunoblotting with antibodies as indicated. *C*, The A549 cells were treated with TGFβ for 48 h and stained for vimentin (*red*), lamin B1 (*green*), and DNA (*blue*). The percentage of the cells with a crumpled or lobulated nucleus was measured (n ≥ 320). Values (means ± SD) were from three independent experiments. ∗∗∗*p* < 0.001.

The expression of vimentin is a hallmark of EMT, which is known to be important for cancer cell migration and invasion (22, 23, 24). However, it is not clear whether VIFs are involved in nuclear dysmorphia during EMT. To examine this possibility, vimentin gene was knocked out in human lung cancer A549 cells, which has been wildly used as an *in vitro* model for TGFβ-induced EMT (7, 43, 44, 45). The depletion of vimentin did not affect TGFβ-induced activation of Smad 3 (Fig. 5*A*), a core transcription factor involved in the TGFβ signaling (46). In addition, the depletion of vimentin did not affect some characteristic events during EMT, including increased expression of fibronectin and N-cadherin and the suppression of E-cadherin (Fig. 5*B*). However, TGFβ stimulation increased the expression of vimentin and thereby their perinuclear accumulation, concomitant with increased nuclear dysmorphia (Fig. 5*C*). The depletion of vimentin largely prevented TGFβ from inducing nuclear dysmorphia in A549 cells (Fig. 5*C*). These results support that the increased expression and assembly of vimentin is involved in nuclear dysmorphia during EMT.

### VIF-mediated nuclear dysmorphia is associated with genome instability

**Figure 6:**
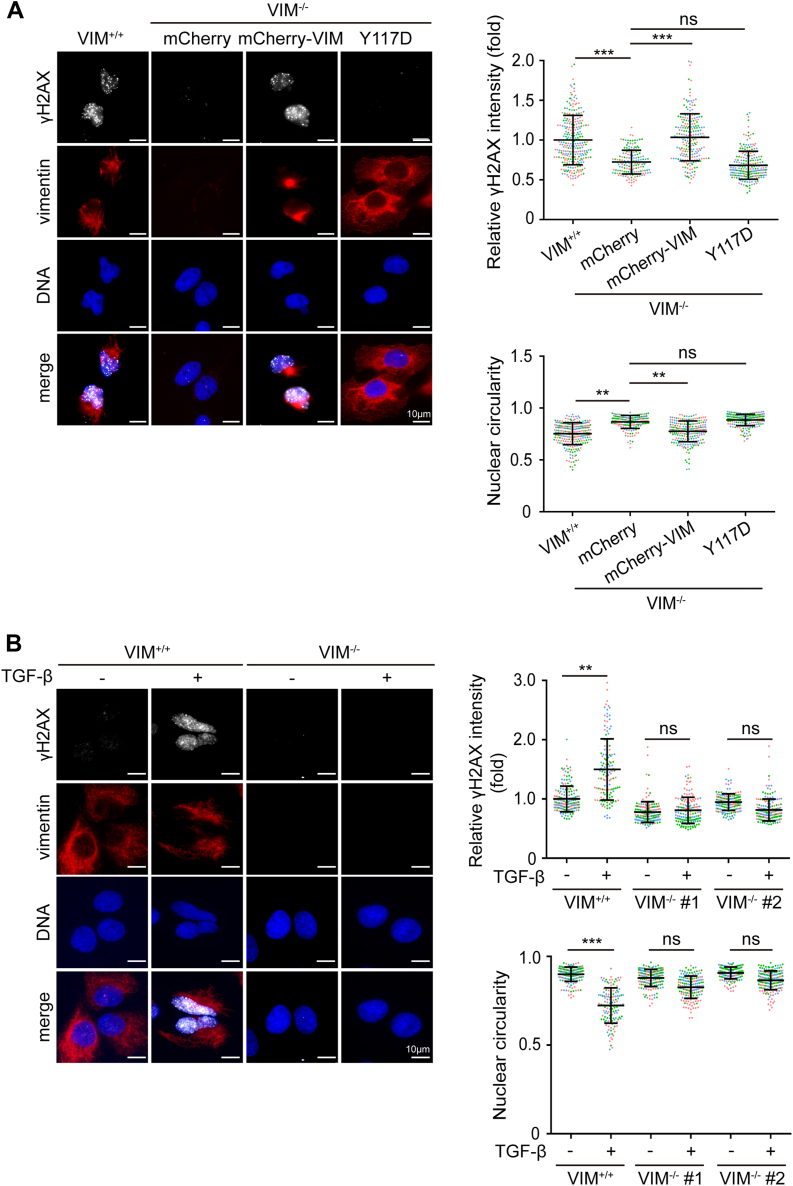
**VIF-induced nuclear dysmorphia is associated with increased DNA damage.***A*, MDA-MB-231 VIM^−/−^ cells were infected by the lentiviruses expressing mCherry, mCherry-vimentin, or the Y117D mutant for 24 h and selected with neomycin for 5 days. The cells were stained for γH2AX (*white*), mCherry-vimentin (*red*), and DNA (*blue*). Representative images are shown. Scale bars, 10 μm. The fluorescence intensity of γH2AX was measured (n ≥ 230) and expressed as –fold relative to the control MDA-MB-231 VIM^+/+^ cells. The nuclear circularity of the cells was determined (n ≥ 230). *B*, A549 VIM^+/+^ and VIM^−/−^ cells were treated with (+) or without (−) TGFβ for 48 h. The cells were stained for γH2AX (*white*), vimentin (*red*), and DNA (*blue*). Representative images are shown. Scale bars, 10 μm. The fluorescence intensity of γH2AX was measured (n ≥ 172) and expressed as –fold relative to the A549 VIM^+/+^ without TGFβ treatment. The nuclear circularity of the cells was determined (n ≥ 172). Data information: values (means ± SD) were from three independent experiments. ∗∗*p* < 0.01, ∗∗∗*p* < 0.001.

**Figure 7:**
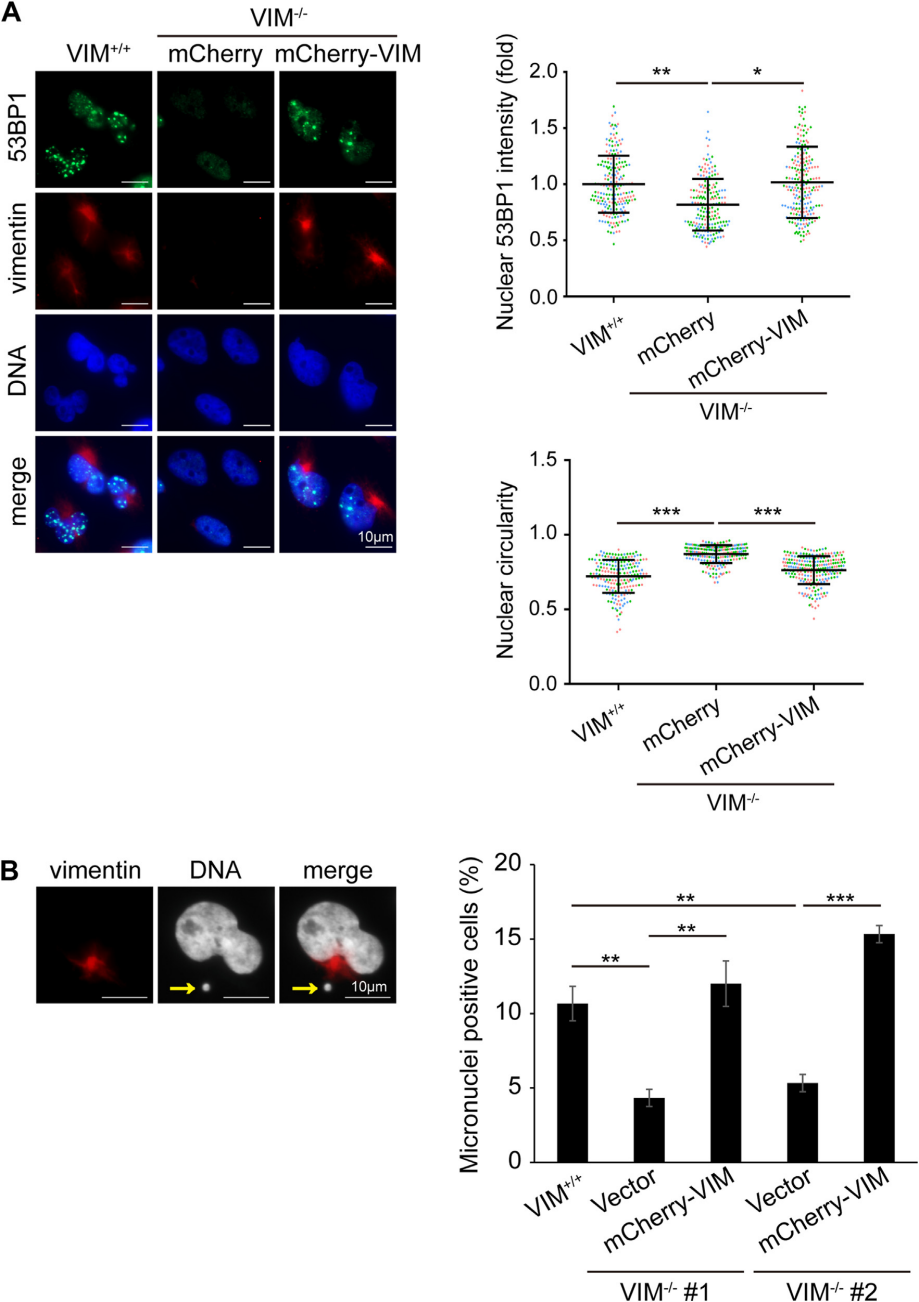
**VIF-mediated nuclear dysmorphia is associated with increased 53BP1 and micronuclei in MDA-MB-231 cells.***A*, MDA-MB-231 VIM^−/−^ cells were infected with the lentiviruses expressing mCherry (vector) or mCherry-vimentin for 24 h and selected with neomycin for 5 days. The cells were stained for 53BP1 (*green*), vimentin (*red*), and DNA (*blue*). Scale bars, 10 μm. The fluorescence intensity of 53BP1 was measured (n ≥ 204) and expressed as –fold relative to the control MDA-MB-231 VIM^+/+^ cells. The nuclear circularity (4π x area/perimeter^2^) of the cells was determined (n ≥ 204). *B*, two vimentin-deficient MDA-MB-231 cell clones (VIM^−/−^ #1 and #2) were transiently transfected with the plasmids encoding mCherry and mCherry-vimentin for 24 h and stained for vimentin (*red*), and DNA (*white*). Scale bars, 10 μm. The arrows indicate micronucleus. The percentage of the transfection-positive cells with micronuclei (size > 1 μm) was measured (n ≥ 329). Values (means ± SD) were from three independent experiments. ∗*p* < 0.05, ∗∗*p* < 0.01, ∗∗∗*p* < 0.001.

Nuclear dysmorphia has been shown to be associated with genome instability (7, 12). To examine whether this is the case for VIF-mediated nuclear dysmorphia, the extent of DNA damage was measured by immunofluorescence staining for γH2AX, a molecular marker for DNA damage (47). We found that the intensity of γH2AX in MDA-MB-231 VIM^−/−^ cells was significantly lower than that in their control counterparts (Fig. 6*A*). The re-expression of mCherry-vimentin, but not the Y117D mutant, into MDA-MB-231 VIM^−/−^ cells devastated their nuclear shape, accompanied by increased γH2AX (Fig. 6*A*). In A549 cells, TGFβ induced nuclear dysmorphia and increased γH2AX, which was suppressed by the depletion of vimentin (Fig. 6*B*). In MCF7 cells, nuclear dysmorphia caused by ectopic expression of vimentin also led to increased γH2AX (Fig. S10). Like γH2AX, the intensity of 53BP1, a key mediator involved in DNA double-strand break repair (48), was correlated with vimentin expression and nuclear dysmorphia in MDA-MB-231 cells (Fig. 7*A*). In addition, we noted that ∼10% of MDA-MB-231 cells contained micronuclei, which was significantly decreased by vimentin depletion (Fig. 7*B*).

To examine whether the transcription of certain DNA repair genes are affected by VIFs in MDA-MB-231 cells, we carried out RNA-sequencing analysis for MDA-MB-231 VIM^+/+^ and VIM^−/−^ cells and found that the transcript levels of XRCC5/Ku80 and DCLRE1B are higher in VIM^−/−^ cells (Fig. S11*A*). Both XRCC5/Ku80 and DCLRE1B are known to be involved in DNA repair (38, 49). The increased mRNAs of XRCC5/Ku and DCLRE1B in VIM^−/−^ cells were further validated by quantitative real-time polymerase chain reaction (PCR) (Fig. S11*B*). These results support that VIF-mediated nuclear dysmorphia can induce downregulation of DNA repair proteins and cause defects in the DNA repair mechanism, leading to genome instability.

## Discussion

Nuclear dysmorphia has been shown to be associated with the malignancy of certain tumors. However, its underlying mechanism and biological significance in tumorigenesis remain elusive. In the present study, we show that VIFs are crucial for nuclear dysmorphia in breast cancer MDA-MB-231 cells (Fig, 2, *A* and *B*) and pancreatic cancer PANC1 cells (Fig. S2). In addition, VIFs are necessary for nuclear dysmorphia during TGFβ-induced EMT in lung cancer A549 cells (Fig. 5). Moreover, ectopic expression of vimentin is sufficient to induce nuclear dysmorphia in breast cancer MCF7 cells (Figs. 2, *C* and *D*; S5 and S6). Therefore, our results in this study support that VIFs play an important role in cancer cell nuclear dysmorphia, at least, in some cancer cells. To the best of our knowledge, this is the first report to claim VIFs with a role in cancer cell nuclear dysmorphia. Actually, the role of VIFs in nuclear deformation has been reported in other types of cells. For example, VIFs are involved in the formation of nuclear blebs and grooves in mouse embryonic fibroblasts (37) and regulate nuclear segmentation in neutrophils (50). However, VIFs may also play a protective role against severe nuclear deformation and nuclear rupture under certain circumstances. In mouse embryonic fibroblasts, VIFs can form a cage-like network structure (51) and protect against nuclear rupture and DNA damage during migration through small pores (52). Nevertheless, such a cage-like structure of VIFs was not observed in the cancer cell lines employed in this study.

It is worth noting that ectopic expression of vimentin did not always induce nuclear dysmorphia in the cell lines we examined (data not shown). This suggests that the rigidity of the nuclear lamina by itself may be the most critical factor for nuclear shape. Only when the rigidity of the nuclear lamina is reduced to a certain extent, the nuclear shape can be affected by mechanical forces that directly apply to the nucleus. The rigidity of the nuclear lamina is mainly dependent on its structural integrity, which can be affected by the expression level, post-translational modification, and mutation of lamins. Recently, it was reported that upon TGFβ stimulation, AKT2 is activated and directly phosphorylates lamin A mainly at Ser390 (7). The increased phosphorylation of lamin A at Ser390 has an adverse effect on lamin A assembly, leading to a “relaxed” nuclear lamina. In this study, we further demonstrate that TGFβ-induced VIFs may exert mechanical force on the nucleus and cause nuclear dysmorphia during TGFβ-induced EMT (Fig. 5). Besides, it is already known that the laminopathy-associated nuclear dysmorphia results from *LMNA* gene mutations, which lead to loss of the nuclear lamina integrity (11). But, what kind of mechanical forces directly apply to the nucleus to cause nuclear dysmorphia in the cells of patients with laminopathies remains unclear. It was reported that the cells derived from progeria patients (a severe type of laminopathy) have increased formation of actin filaments (10), rendering it possible that actomyosin-mediated contractility may cause nuclear dysmorphia in those progeria cells. Experiments are in progress to examine this possibility.

In this study, we show that perinuclear accumulation of VIFs is important for them to induce nuclear dysmorphia (Fig. 4). Since VIFs are the major type of cytoplasmic intermediate filaments expressed in cancer cells, their perinuclear accumulation could directly compress the nucleus and thereby cause its deformation, especially when the nuclear rigidity is reduced. It is not known whether other types of intermediate filaments have such a nucleus-deforming property similar to VIFs. In this study, we demonstrate that keratin intermediate filaments are not involved in nuclear dysmorphia in MDA-MB-231 cells (Figs. S3 and S4). Moreover, we found that plectin was apparently co-localized with VIFs at the perinuclear region of MDA-MB-231 cells (Fig. 4), supporting that plectin serves as a cytoskeletal linker mainly for the intermediate filaments (34). Since plectin has been shown to be overexpressed in some cancers (53, 54), it is possible that when cancer cells simultaneously overexpress both vimentin and plectin, they may have more VIFs accumulated at the perinuclear region and thereby tends to undergo nuclear dysmorphia. Although high expression of vimentin is associated with poor recurrence-free survival of breast cancer patients (Fig. S12), it is unclear whether simultaneous high expression of both vimentin and plectin leads to a worse scenario.

Previous reports indicate that nuclear dysmorphia is often accompanied by mis-localization or degradation of DNA repair proteins, leading to genome instability. For example, deletion of lamin A leads to mis-localization of ATR (16, 17) and degradation of 53BP1 (14, 15). Likewise, TGFβ-induced nuclear dysmorphia also displays similar defects in ATR and 53BP1 (7). In this study, we found that VIF-induced nuclear dysmorphia is also associated with defects in DNA repair, manifested by increased levels of γH2AX and 53BP1 in the nucleus (Figs. 6 and 7), both of which serve as molecular markers for DNA double-strand breaks (47, 48). However, VIF-induced nuclear dysmorphia did not appear to cause the mis-localization of ATR in MDA-MB-231 cells. It may be simply because of the cell type-dependent effect, or it may raise an intriguing possibility that nuclear dysmorphia induced by different mechanisms may disturb DNA repair by affecting different DNA repair proteins. In this study, our results suggest that VIF-induced nuclear dysmorphia of MDA-MB-231 cells induces downregulation of XRCC5/Ku80 and DCLRE1B (Fig. S11). XRCC5, also known as Ku80, together with XRCC6 (Ku70) constitutes the XRCC5/XRCC6 heterodimer (Ku80/Ku70), which is a DNA-dependent protein kinase complex (55, 56, 57). The XRCC5/XRCC6 dimer binds to DNA double-stranded break ends and serves as an essential component of DNA nonhomologous end-joining repair (38). The DCLRE1B gene encodes a DNA exonuclease involved in the repair of DNA interstrand crosslinks and DNA double-strand breaks, as well as in the stabilization of stalled replication forks and S-phase checkpoint activation. DCLRE1B is one of several evolutionarily conserved genes involved in the repair of interstrand cross-links (49). The work by (30) reported that the expression of vimentin in MCF7 cells alters their gene expression profiles, among which the expression of the MMS22L gene was decreased. MMS22L is the abbreviation for methyl methanesulfonate-sensitivity protein 22-like, which is crucial in protecting genome integrity during DNA replication by preventing DNA damage and maintaining efficient homologous recombination (58, 59).

Taken together, the scenario we proposed for cancer cell nuclear dysmorphia is that: (i) Decreased rigidity of the nuclear lamina caused by defects in the nuclear lamina organization (*e.g.* decreased expression, increased phosphorylation and/or mutation of lamins) can render the nucleus susceptible for deformation by mechanical forces that directly apply on the nucleus. (ii) The forces that affect nuclear shape can arise from both outside and inside the nucleus. Outside the nucleus, peri-nuclear accumulation of vimentin filaments can drive cancer cell nuclear dysmorphia. Inside the nucleus, chromatin compaction *via* increased heterochromatin can confer rigidity to the nucleus (7, 13). (iii) Nuclear dysmorphia may cause mis-localization and/or downregulation of certain DNA repair proteins, and thereby abrogate the DNA repair mechanism, leading to a loss of genome integrity.

**Figure 8:**
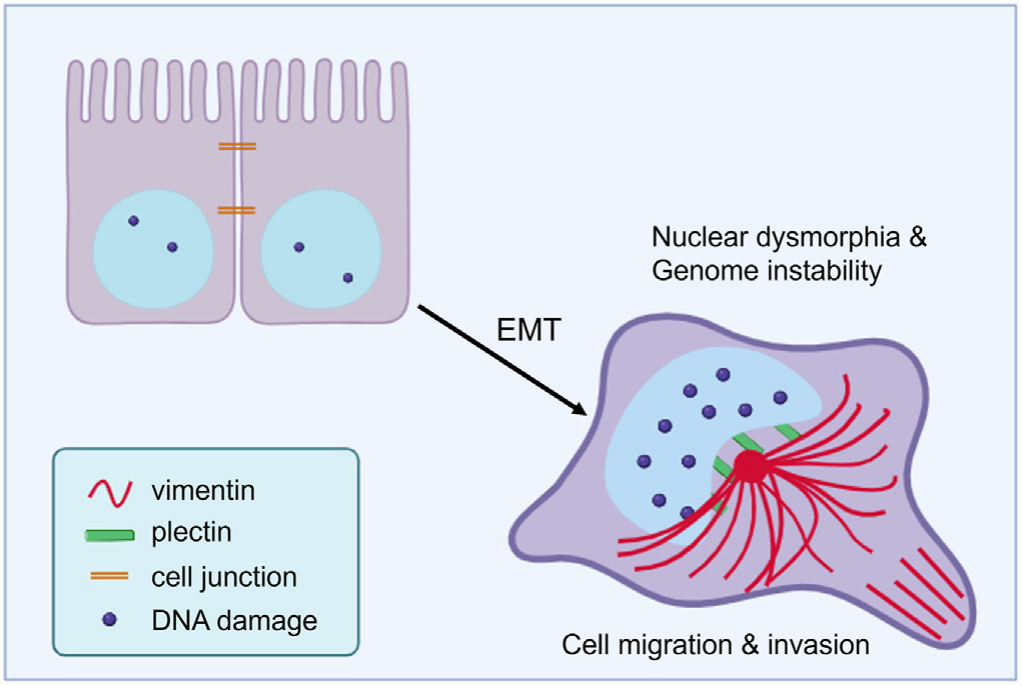
**The scheme depicts the role of vimentin in tumor progression.** Previous studies have shown that vimentin contributes to tumor progression through their effects on cell integrity, cell adhesion, cell motility, and invasion. The present study unveils an important role of vimentin in cancer cell nuclear dysmorphia and genome instability, which further enhances our understanding to the spectrum of vimentin’s biological effects as well as how VIFs contribute to tumorigenesis.

VIFs have long been used as a hallmark of EMT. Extensive studies have already shown that VIFs are associated with tumor malignancy through their effects on cell integrity, cell adhesion, cell motility, and invasion (22, 23, 24). The present study unveils an important role of VIFs in cancer cell nuclear dysmorphia and genome instability, which further enhances our understanding of the spectrum of VIF’s biological effects as well as how VIFs contribute to tumorigenesis (Fig. 8). Other and our studies suggest that the strategies to inhibit vimentin’s expression, assembly, or perinuclear accumulation in cancer cells may ameliorate their malignancy.

## Experimental procedures

### Antibodies and reagents

The mouse monoclonal anti-lamin A (clone 133A2) and rabbit polyclonal anti-lamin B1 anti-N-cadherin, and anti-lamin A/C pS392 antibodies were purchased from Abcam. The rabbit polyclonal anti-vimentin and anti-mCherry antibodies were purchased from GeneTex. The customized rabbit polyclonal anti-lamin A/C antibody was generated by GeneTex. The mouse monoclonal anti-vimentin (clone V9), anti-vimentin (clone VIM-13.2), anti-keratin 8 (clone M20), anti-keratin 18 (clone CY90), anti-α-tubulin (clone DM1A), anti-FLAG (clone M2), anti-β-actin (clone AC-15) and rabbit polyclonal anti-FLAG, anti-trimethyl-histone H3 (Lys27), and anti-fibronectin antibodies were purchased from Sigma-Aldrich. The mouse monoclonal anti-E-cadherin (clone 34) and anti-53BP1 (clone 19) antibodies were purchased from BD Biosciences. The mouse monoclonal anti-Smad2/3 (clone C-8) and anti-plectin (clone 10F6) antibodies were purchased from Santa Cruz Biotechnology. The rabbit polyclonal anti-Smad3 pS423/425 and phalloidin (conjugated with Alexa Fluor-488) were purchased from Invitrogen. The mouse monoclonal anti-H2AX pS139 (clone JBW301) and rabbit polyclonal anti-lamin A/C pS390 and anti-lamin A/C pS404 antibodies were purchased from Merck Millipore. The rabbit polyclonal anti-lamin A/C pS22 and rabbit monoclonal anti-STING (clone D2P2F) and anti-STING pS366 (clone E9A9K) antibodies were purchased from Cell Signaling Technology. Recombinant human TGFβ was purchased from R&D Systems. 3-Deazaneplanocin-A (DZNep) was purchased from Selleckchem. Cytochalasin D, blebbistatin, ML7, and nocodazole were purchased from Sigma-Aldrich.

### Plasmids

For FLAG-vimentin, the human vimentin cDNA was cloned into the pFLAG-CMV-5b plasmid using BamHI and EcoRI sites. The plasmids pmCherry-vimentin series (WT, S39D, S72D, Y117D, and Y117F) were described previously (32, 60).

### Cell culture and transfection

The human cell lines used in this study were obtained from the American Type Culture Collection and periodically examined for *mycoplasma* contamination. The cell lines including MDA-MB-231, PANC-1, MCF7, and HEK293T were cultured in DMEM medium supplemented with 10% FBS. A549 cells were cultured in RPMI1640 medium supplemented with 10% FBS. TGFβ (5 ng/ml) was used to induce EMT of A549 cells. The inhibitors including cytochalasin. D (150 nM), blebbistatin (25 μM), ML7 (50 μM), Y27632 (50 μM), nocodazole (10 uM), and DZNep (5 uM) were used as described in the figure legends. Lipofectamine 2000 (Invitrogen) was used to perform transient transfection.

### Lentivirus production and infection

The lentiviral expression system was provided by the National RNAi Core Facility, Academia Sinica, Taiwan. To produce the lentiviruses, HEK293T cells were co-transfected with 0.25 μg pMD.G, 2.25 μg pCMV-ΔR8.91, and 2.5 μg pLAS3w-pNeo-mCherry-vimentin, pLKO.1-puro-shPlectin, pLKO.1-puro-shVimentin, pLKO.1-puro-shKeratin 8, or pLKO.1-puro-shKeratin 18. The target sequences of shRNAs used in this study are: 5′-GCCTTCCATGTCGGCCTCTAA-3’ (shPlectin #1), 5′-CGATGAGGAGATGAACGAGAT-3’ (shPlectin #2), 5′-CCTCTTCGATGAGGAGATGAA-3’ (shPlectin #3), 5′-GCTAACTACCAAGACACTATT-3’ (shVimentin #1), 5′-GCAGGATGAGATTCAGAATAT-3’ (shVimentin #2), 5′-TCGAAGCAACATGGACAACAT-3’ (shKeratin 8 #1), 5′-GCAGCTATATGAAGAGGAGAT-3’ (shKeratin 8 #2), 5′-CAGATTGACAATGCCCGTCTT-3’ (shKeratin 18 #1), and 5′-GATGACACCAATATCACACGA-3’ (shKeratin 18 #2). After 3 days, the medium containing the viral particles was collected and stored at −80 °C. The cells were infected by lentiviruses for 24 h and selected with neomycin (1 μg/ml) or puromycin (2 μg/ml) for 5 days.

### CRISPR/Cas9-mediated gene editing and genotyping

Vimentin, plectin, and keratin 8 genes were edited by co-expression of Cas9 (Addgene plasmid #41815) and gRNA (Addgene plasmid #41824) containing the target sequences for vimentin (5′-GCTCCTCTGCCGTGCGC-3′), plectin (5′-GAAGAAAACCTTCACCAAGTGG-3′), or keratin 8 (5′-CACCTTCTCCACCAACTACCGG-3′). The cells deficient in vimentin or plectin were obtained through clonal propagation from a single cell. Individual clones were validated by immunofluorescence staining and immunoblotting. For genotyping, genomic DNA of the cells deficient in vimentin, plectin, or keratin 8 were extracted and then subjected to PCR with the following primers: 5′-CTGGATCCAGTCCTCTGCCACTCTCGCT-3′ (forward primer for vimentin), 5′-GAGAATTCTCGGCCAGCAGGATCTTATT-3′ (reverse primer for viemntin), 5′-TGGGATCCCAGGACCCTCCCATTTCCTC-3′ (forward primer for plectin) 5′-GGGAATTCCCACGGAGGCACCTACCTCT-3′ (reverse primer for plectin), 5′-TCGGATCCGAGTGGGCTCCAGGGTTGGA-3′ (forward primer for keratin 8), and 5′-GGGAATTCCCTTGTCTATGAAGGAGGCA-3′ (reverse primer for keratin 8). The PCR products were cloned and sequenced. The display of DNA sequences was acquired by the Vector NTI Advance 7.1 software.

### RNA sequencing

Total RNAs of MDA-MB-231 VIM^+/+^ and VIM^−/−^ cells were extracted by TRIzol reagent (Invitrogen). RNA purity and quantification were examined using SimpliNano - Biochrom Spectrophotometers (Biochrom). RNA degradation and integrity were monitored by Qsep 100 DNA/RNA Analyzer (BiOptic Inc). The extracted RNA was entrusted to Biotools Company (Taiwan) for library preparation and RNA sequencing. Sequencing libraries were generated using KAPA mRNA HyperPrep Kit (KAPA Biosystems, Roche) and high throughput sequencing was performed using Illumina NovaSeq6000 platform. The read pairs from MDA-MB-231 VIM^+/+^ and VIM^−/−^ cells were aligned to the reference genome (*Homo sapiens* genome assembly NCBI36, hg18) by the HISAT2 software. The differentially expressed genes analysis was performed using DESeq2. The human DNA repair genes were from the Molecular Signatures Database (MSigDB), a collection of annotated gene sets for use with Gene Set Enrichment Analysis (GSEA) software.

### Quantitative real-time PCR

Total RNA was extracted by the Quick-RNA MiniPrep kit (Zymo Research). The first strand cDNA was synthesized by the RevertAid First Strand cDNA Synthesis kit (Thermo Fisher Scientific). Quantitative real-time PCR was performed by using the SYBR Green PCR Master Mix (Bio-Rad Laboratories) with the primers and analyzed using the QuantStudio 3 Real-Time PCR system (Thermo Fisher Scientific). The primer sequences used in this study are: RPL19 (encoding ribosomal protein L19 as an internal control), 5′-GAAATCGCCAATGCCAACTC-3′ (sense) and 5′-TCCTTGGTCTTAGACCTGCG-3′ (antisense); XRCC5, 5′-GTGCGGTCGGGGAATAAGG-3′ (sense) and 5′-GGGGATTCTATACCAGGAATGGA-3′ (antisense); DCLRE1B, 5′-ACCTCTTGCATCGTCACCTAC-3′ (sense) and 5′-CTAGGGGTAATACATGGCTCTCA-3′ (antisense).

### Immunofluorescence staining, microscopy, and image analysis

Cells were fixed with phosphate-buffered saline containing 4% paraformaldehyde for 30 min, and subsequently permeabilized with 0.5% Triton X-100 for 15 min. The cells were stained with primary antibodies for 1 h and followed by incubation with Alexa Fluor 488- or 546-conjugated secondary antibodies or phalloidin for 1 h. Coverslips were mounted on the slides with Anti-Fade Dapi-Fluoromount-G (Southern Biotech). The images in Figures 1, *A* and *B*, 2, *B* and *D*, 3, *B* and *E*, 5*C*, S2*C*, S5*B*, S9, *A* and *B* were acquired using a Carl Zeiss LSM880 confocal microscope imaging system with Zeiss Plan-Apochromat 40x and 63x/NA 1.4 oil immersion objectives. The images in Figures 1*D*, 4*A*, 6, *A* and *B*, 7, *A* and *B*, S3*B*, S4*C*, S6, S7*B*, S9*C*, S10, *A* and *B* were acquired using a Carl Zeiss Apotome2 microscope system, equipped with Zeiss Plan-Apochromat 40x and 63x/NA 1.4 oil immersion objectives and a camera (ORCA-Flash4.0 V2; Hamamatsu). The representative images were cropped by Photoshop CS6 (Adobe) and assembled into figures by Illustrator CS6 (Adobe). The angle of concaves in Figure 1*B* and the fluorescence intensities in Figures 1, *A* and *C*, 6, *A* and *B*, 7*A*, S9*C*, S10, *A* and *B* were quantitated by the ZEN2 software (Carl Zeiss). To measure the perinuclear accumulation of VIFs in Figures 4*C* and S7*C*, the distribution of VIFs within a 25 × 25 μm perinuclear area was defined as perinuclear accumulation.

### Immunoblotting

Cells were lysed on ice in RIPA buffer (0.1% SDS, 1% sodium deoxycholate, 1% NP-40, 150 mM NaCl, 50 mM Tris-HCl, 1 mM EDTA, pH 7.4) and the protein concentration was measured by the Bradford protein assay reagent (Bio-Rad). The samples were subjected to SDS-polyacrylamide gel electrophoresis and transferred to nitrocellulose membranes. Membranes were incubated with the primary antibodies and subsequently with secondary antibodies conjugated to horseradish peroxidase or IR-780/680 iodide fluorescence dye, and then scanned by the luminescence imaging system (LAS-4000; Fujifilm) or the Odyssey CLx Imaging System (LI-COR Biosciences).

### Densitometric quantitation and statistics

Densitometric quantitation of the scanned images was performed using the NIH Image J software or Odyssey CLx Imaging System (LI-COR Biosciences). The two-tailed Student’s *t* test was used to determine whether the differences between experimental values were significant. *p* < 0.05 was considered statistically significant. The number of cells counted for each experiment and the number of experiments performed were described in the figure legends. Adobe Illustrator CS6 was used for preparing the figures.

## Data availability

The RNA sequencing data was deposited in the Gene Expression Omnibus (GEO) database under accession number GSE279178.

## Supporting information

This article contains supporting information.

## Conflict of interests

The authors declare that they have no conflicts of interest with the contents of this article.
