# Supplementary material for: Perinuclear assembly of vimentin intermediate filaments induces cancer cell nuclear dysmorphia
Source: J Biol Chem. 2024 Nov 13;300(12):107981. doi: 10.1016/j.jbc.2024.107981 (PMC11647624; doi:10.1016/j.jbc.2024.107981)
Supplement: Supporting information [file mmc1.pdf]

Supplementary Information

Perinuclear assembly of vimentin intermediate filaments induces cancer cell nuclear dysmorphia

Ke-Wei Pan[1] and Hong-Chen Chen[1,2,3*]

[1]Institute of Biochemistry and Molecular Biology, National Yang Ming Chiao Tung University, Taipei 11221, Taiwan

[2]Cancer and Immunology Research Center, National Yang Ming Chiao Tung University, Taipei 11221, Taiwan

[3]Department of Biotechnology and Bioindustry Sciences, National Cheng Kung University, Tainan 701, Taiwan

*Corresponding author. Email: hcchen1029@nycu.edu.tw

Content

Figure S1 ……………………………………………………………………….….2
Figure S2 ……………………………………………………………………..…..3
Figure S3 ……………………………………………………………………..…..4
Figure S4 ……………………………………………………………………..…..5
Figure S5 ……………………………………………………………………..…..6
Figure S6 ……………………………………………………………………..…..7
Figure S7 ……………………………………………………………………..…..8
Figure S8 ……………………………………………………………………..…..9
Figure S9 ………………………………………………………………………10
Figure S10 ……………………………………………………………………...…11
Figure S11 ……………………………………………………………………...…12
Figure S12 ……………………………………………………………………..13

Supplementary Figures

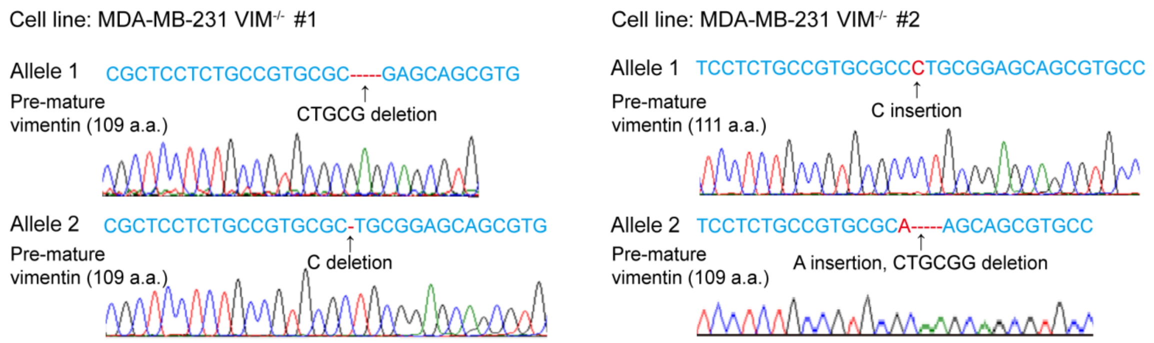

Figure S1. Genotyping for MDA-MB-231 VIM$^{-/-}$ cells. Vimentin gene was edited in MDA-MB-231 cells using the CRISPR/Cas9 system. Two vimentin-deficient cell clones (VIM$^{-/-}$ #1 and #2) were established. The target site of vimentin gRNA was analyzed by genotyping. For genotyping, the genomic DNA of the cells were extracted and then subjected to the polymerase chain reaction with the primers flanked the gRNA-target site. The products of the polymerase chain reaction were cloned and sequenced. The display of DNA sequences was acquired by the Vector NTI Advance 7.1 software. The edited sequences are shown in red.

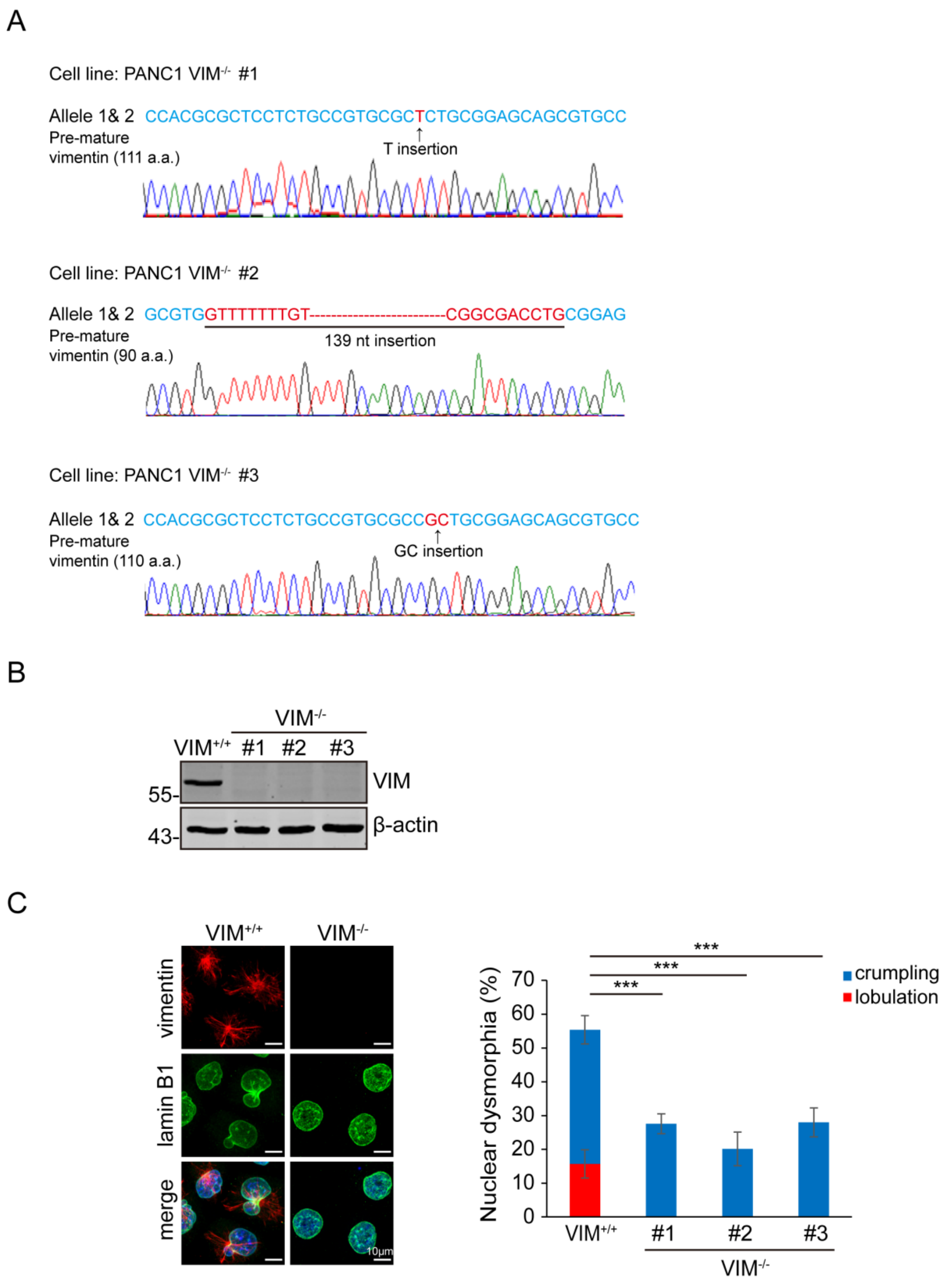

Figure S2. VIFs are important for nuclear dysmorphia in pancreatic cancer PANC1 cells. (**A**) Vimentin gene was edited in PANC1 cells using the CRISPR/Cas9 system. Three vimentin-deficient cell clones (VIM$^{-/-}$ #1, #2, and #3) were established. The target site of vimentin gRNA was analyzed by genotyping. The edited sequences are shown in red. (**B**) An equal amount of whole cell lysates was analyzed by immunoblotting with antibodies as indicated. (**C**) PANC1VIM$^{+/+}$ and VIM$^{-/-}$ cells were stained for vimentin (red), lamin B1 (green), and DNA (blue). Scale bars, 10 μm. The percentage of the cells with a crumpled or lobulated nucleus was measured (n ≥ 321). Values (means ± SD) were from three independent experiments. ****P*<0.001.

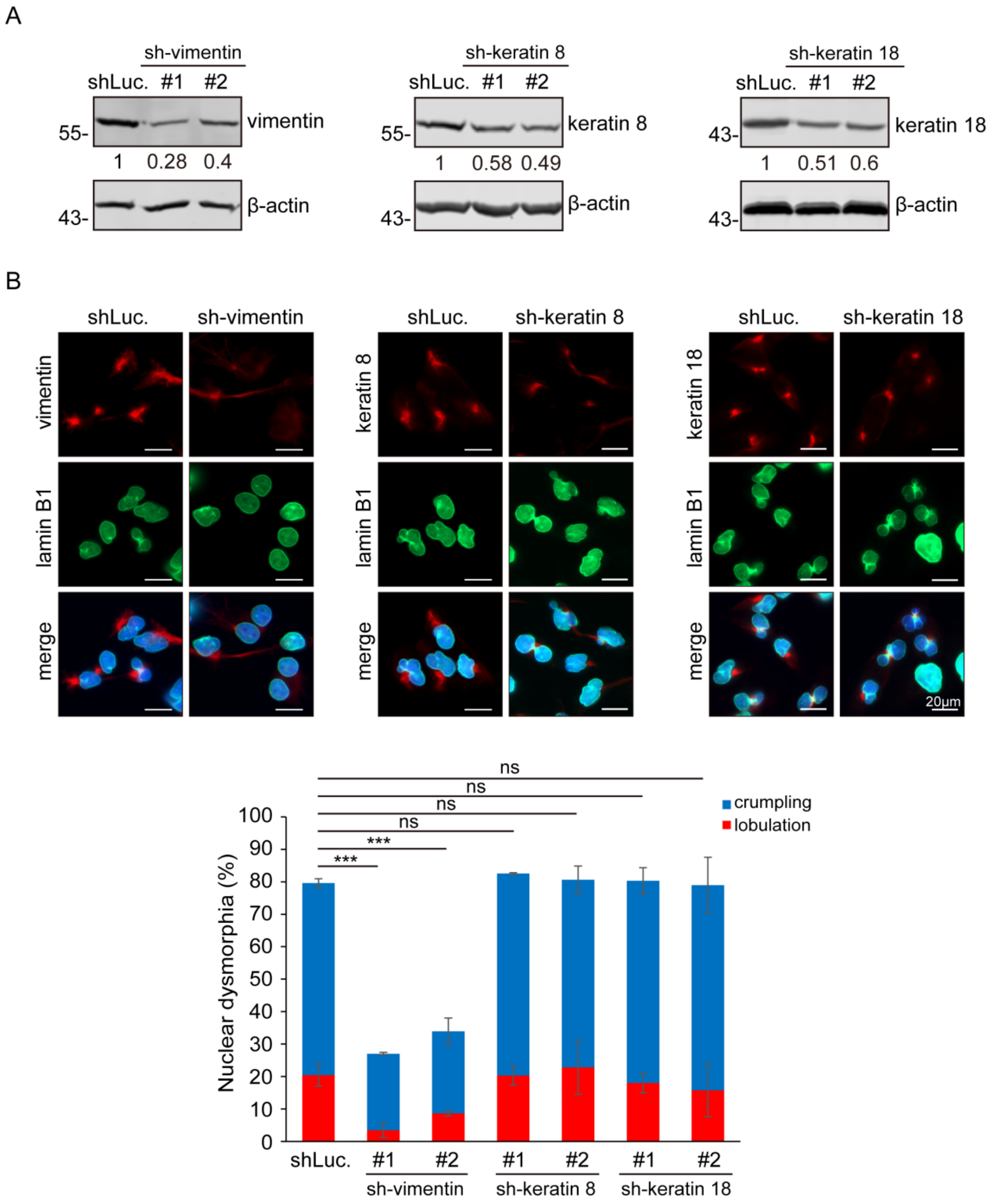

Figure S3. Depletion of keratin 8 or 18 by the shRNA approach does not affect the ratio of nuclear dysmorphia in MDA-MB-231 cells. (A) MDA-MB-231 cells were infected with the lentiviruses encoding shRNAs specific to vimentin (sh-vimentin), keratin 8 (sh-keratin 8), keratin 18 (sh-keratin 18), or luciferase (shLuc) as the control. An equal amount of whole cell lysates was analyzed by immunoblotting with the antibodies as indicated. The level of vimentin, keratin 8 and keratin 18 were measured by immunoblotting and expressed as -fold relative to the level of the shLuc control. **(B)** The cells as described in A were stained for vimentin (red), keratin 8/ keratin 18 (red), lamin B1 (green), and DNA (blue). Representative images are shown. Scale bars, 20 µm. The percentage of the cells with nuclear dysmorphia was measured ($n \geq 315$). Values (means ± SD) were from three independent experiments. ***$P<0.001$.

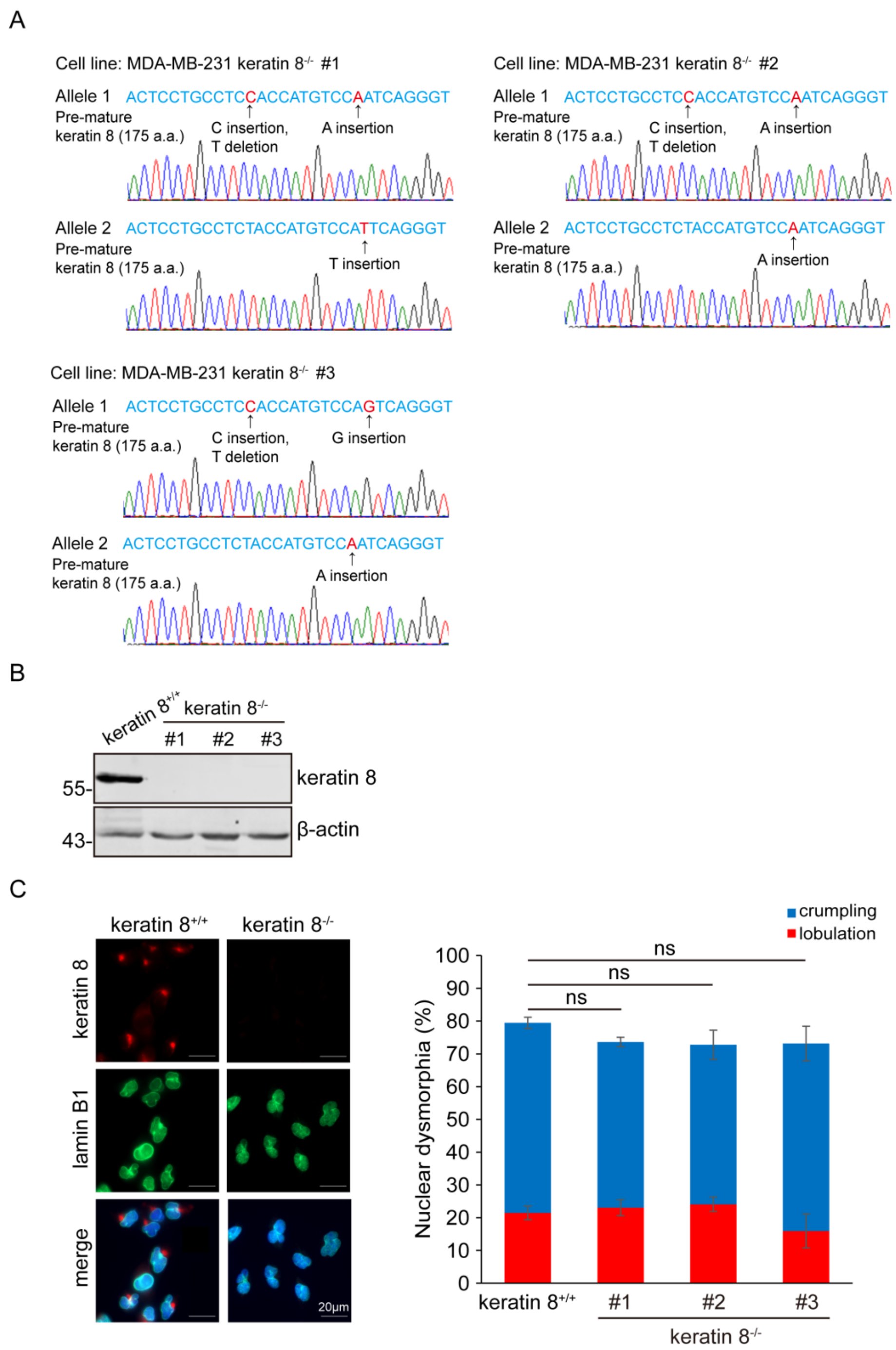

Figure S4. Keratin 8 gene knockout by the CRISPR/Cas9 does not affect the ratio of nuclear dysmorphia in MDA-MB-231 cells. (**A**) Keratin 8 gene was edited in MDA-MB-231 cells using the CRISPR/Cas9 system. Three keratin 8-deficient cell clones (keratin $8^{-/-}$ #1, #2, and #3) were established. The target site of keratin 8 gRNA was analyzed by genotyping. The edited sequences are shown in red. (**B**) An equal amount of whole cell lysates was analyzed by immunoblotting with antibodies as indicated. (**C**) MDA-MB-231 keratin $8^{+/+}$ and keratin $8^{-/-}$ cells were stained for keratin 8 (red), lamin B1 (green), and DNA (blue). Scale bars, 20 μm. The percentage of the cells with a crumpled or lobulated nucleus was measured (n ≥ 303). Values (means ± SD) were from three independent experiments.

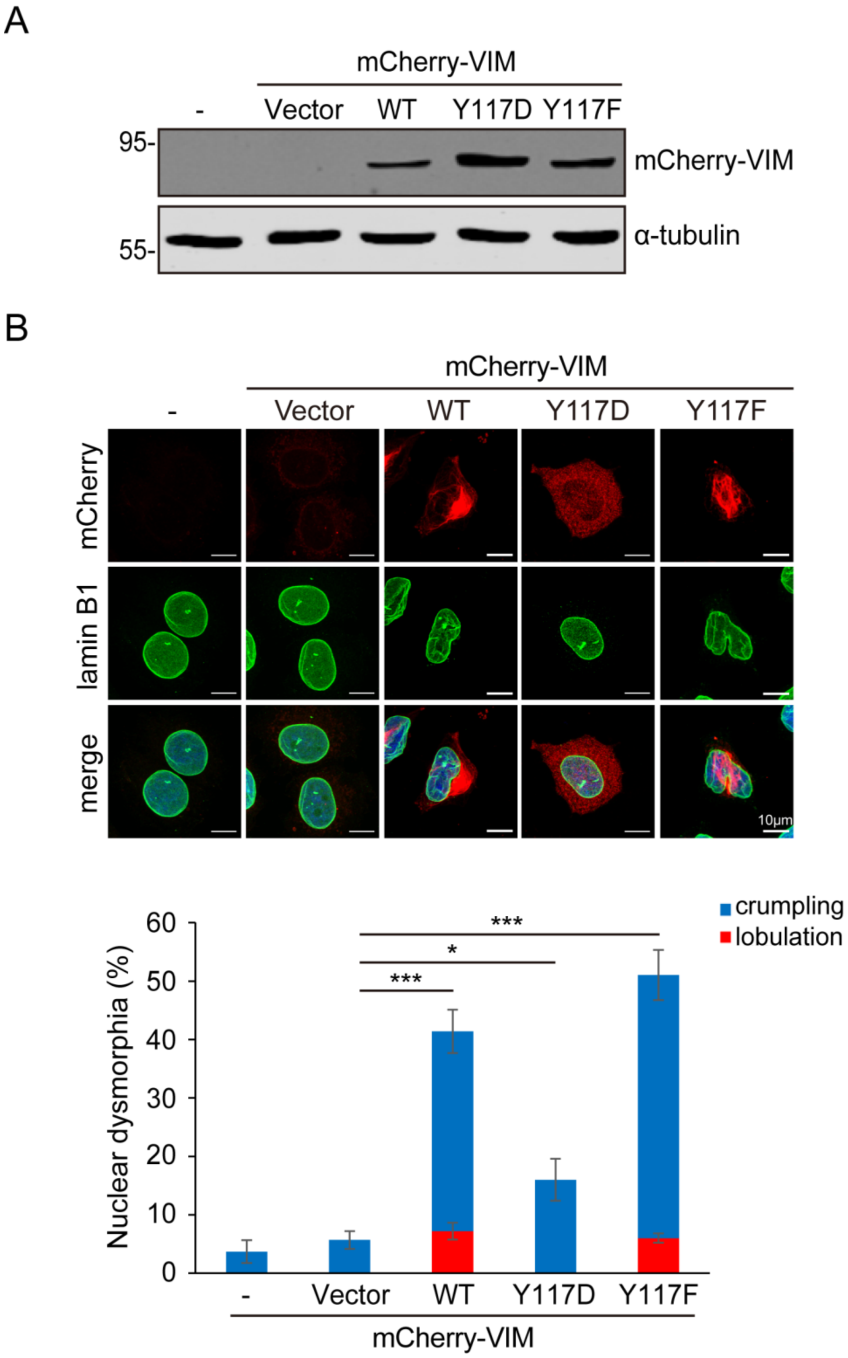

Figure S5. The assembly of vimentin is necessary for inducing nuclear dysmorphia in MCF7 cells. (**A**) MCF7 cells were transiently transfected with the plasmids encoding mCherry (vector), mCherry-vimentin or the Y117 mutants. An equal amount of whole cell lysates was analyzed by immunoblotting with antibodies as indicated. (**B**) The cells as described in A were stained for mCherry (red), lamin B1 (green), and DNA (blue). Scale bars, 10 μm. The percentage of the mCherry-positive cells with a crumpled or lobulated nucleus was measured ($n \geq 314$). Values (means ± SD) were from three independent experiments. *P<0.05, ***P<0.001.

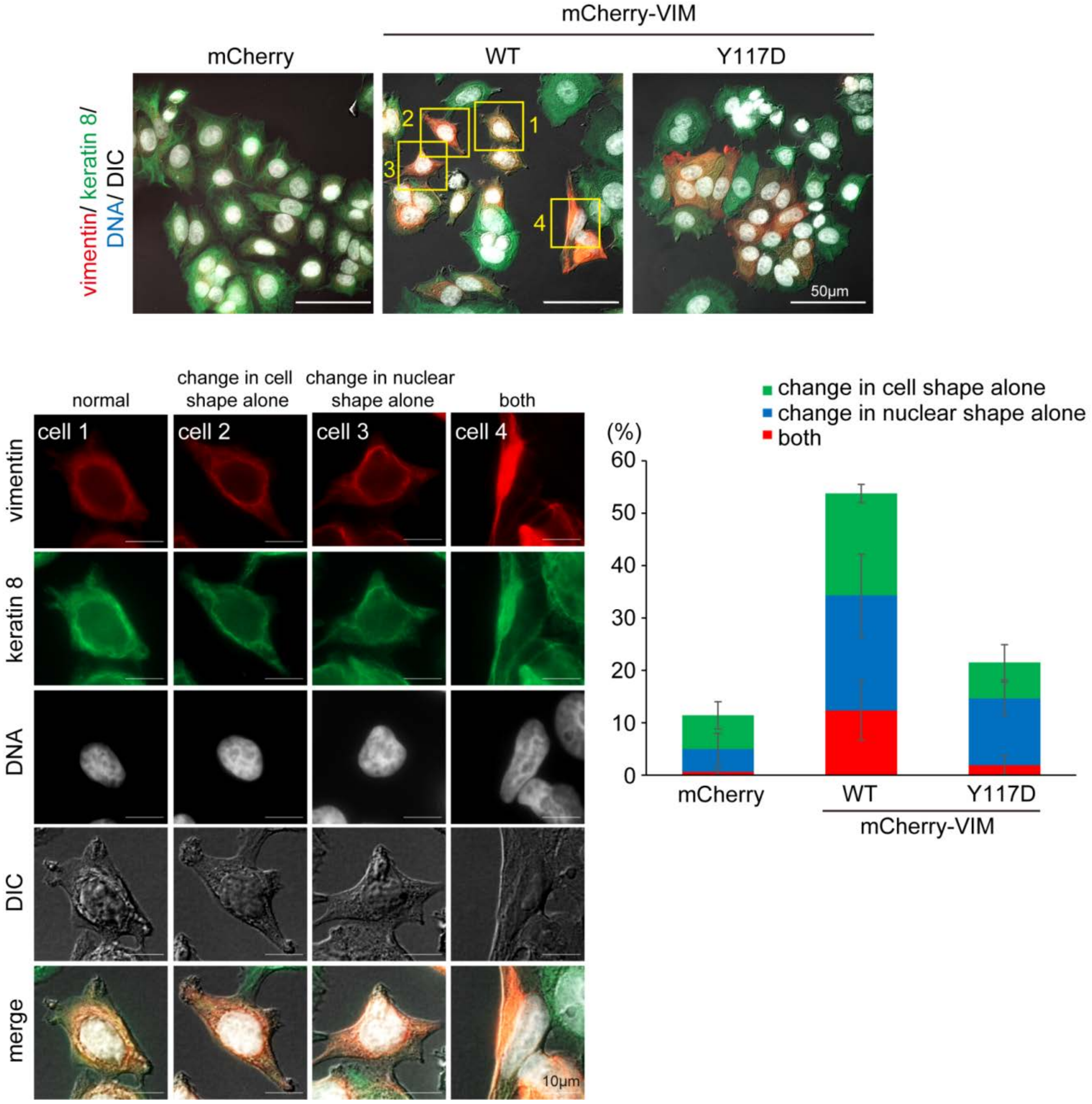

Figure S6. The ectopic expression of vimentin induces changes in cell morphology and nuclear shape in MCF7 cells. MCF7 cells were transiently transfected with the plasmids encoding mCherry (vector), mCherry-vimentin or the Y117D mutant. The cells were stained with antibodies specific to vimentin (red) or keratin 8 (green) and DAPI for DNA (blue). Scale bars, 50 µm. The insets show representative images of the cells with normal cell and nuclear shapes and those with changes in cell shape alone, nuclear shape alone, or both. Scale bars, 10 µm. The cells with the long axis of the cell length ≥ 2.5-fold of the short axis were considered as change in cell shape. Values (means ± SD) were from three independent experiments (n ≥ 267).

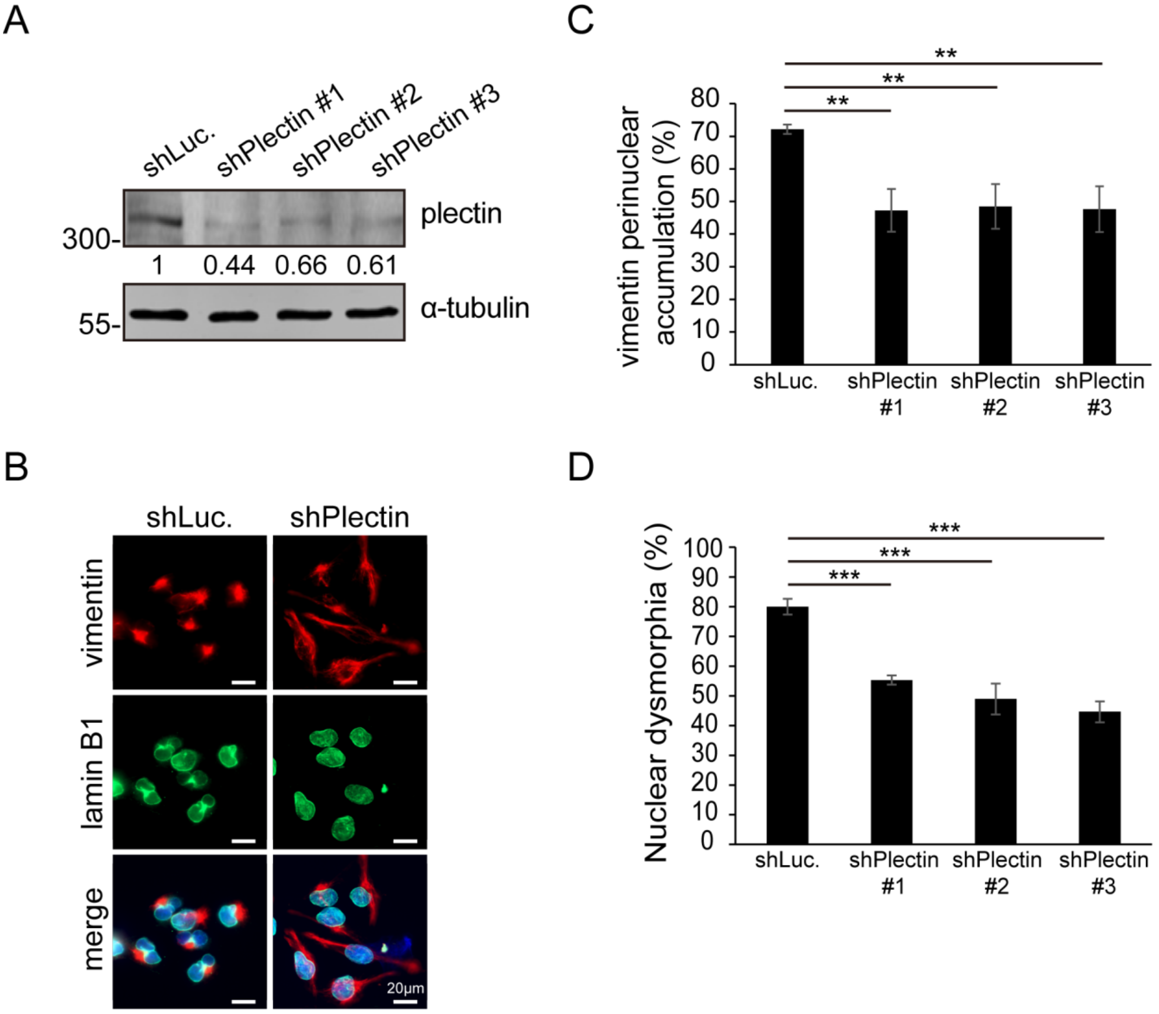

Figure S7. Depletion of plectin by the shRNA approach partially restores the nuclear shape of MDA-MB-231 cells. (**A**) MDA-MB-231 cells were infected with the lentiviruses encoding shRNAs specific to plectin (shPlectin) or luciferase (shLuc) as the control. An equal amount of whole cell lysates was analyzed by immunoblotting with the antibodies as indicated. The level of plectin was measured and expressed as -fold relative to the level of the shLuc control. **(B)** The cells as described in A were stained for vimentin (red), lamin B1 (green), and DNA (blue). Representative images are shown. Scale bars, 20 μm. **(C)** The percentage of the cells with a perinuclear accumulation of vimentin was measured (n ≥ 316). The distribution of vimentin within a 25 x 25 μm perinuclear area is defined as perinuclear accumulation. (**D**) The percentage of the cells with nuclear dysmorphia was measured (n ≥ 209). In *C* and *D*, values (means ± SD) were from three independent experiments. $^{**}P<0.01$, $^{***}P<0.001$.

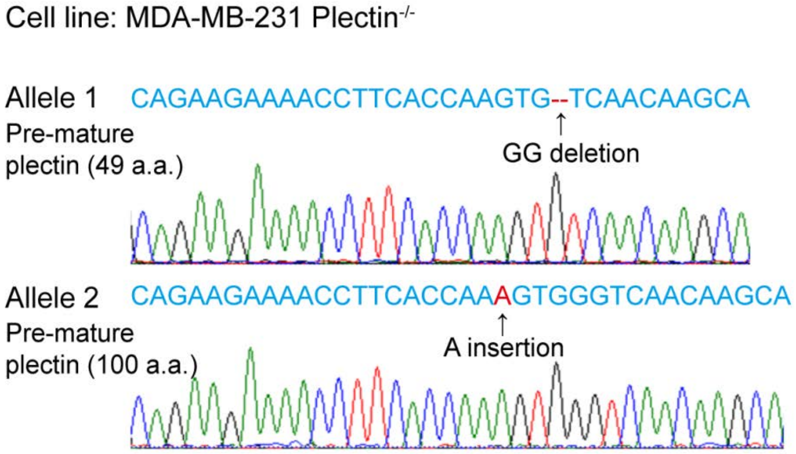

Figure S8. Genotyping for MDA-MB-231 Plectin$^{-/-}$ cells. Plectin gene was edited in MDA-MB-231 cells using the CRISPR/Cas9 system. A plectin-deficient cell clone (Plectin$^{-/-}$) were established. The target site of plectin gRNA was analyzed by genotyping. The edited sequences are shown in red.

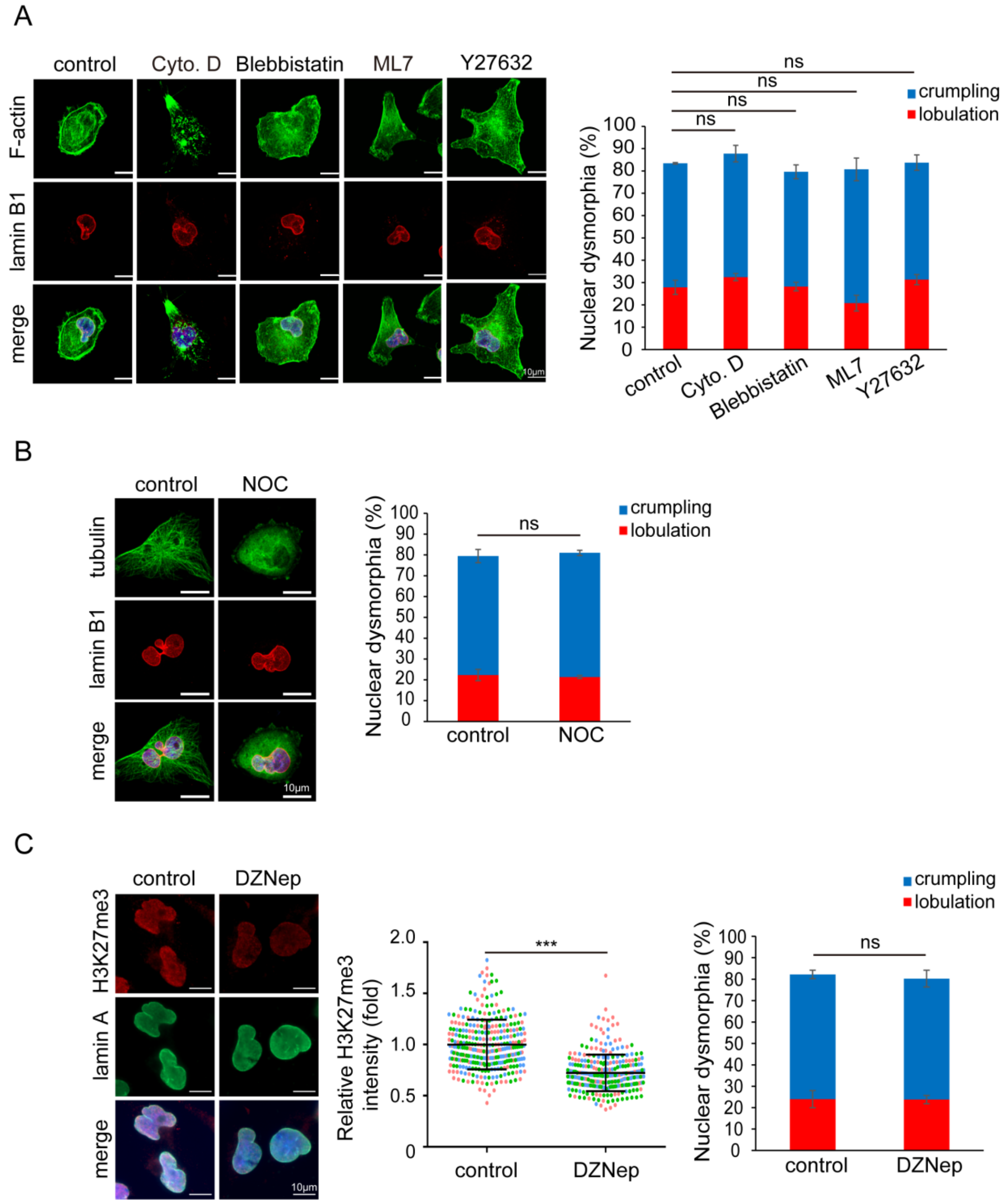

Figure S9. F-actin, microtubule, or heterochromatin is not involved in nuclear dysmorphia of MDA-MB-231 cells. (A) MDA-MB-231 cells were treated with Cyto. D (150 nM), blebbistatin (25 μM), ML7 (50 μM), or Y27632 (50 μM) for 4 h and stained for F-actin (green), lamin B1 (red), and DNA (blue). Scale bars, 10 μm. The percentage of the cells with a crumpled or lobulated nucleus was measured (n ≥ 344). (**B**) MDA-MB-231 cells were treated with nocodazole (10 uM) for 1 h and stained for α-tubulin (green), lamin B1 (red), and DNA (blue). Scale bars, 10 μm. The percentage of the cells with a crumpled or lobulated nucleus was measured (n ≥ 300). (**C**) MDA-MB-231 cells were treated with DZNep (5 uM) for 24 h and stained for H3K27me3 (red), lamin A (green), and DNA (blue). Scale bars, 10 μm. The fluorescence intensity of H3K27me3 was measured (n ≥ 316) and expressed as –fold relative to the control. The percentage of the cells with a crumpled or lobulated nucleus was measured (n ≥ 331). Values (means ± SD) were from three independent experiments. ****P*<0.001.

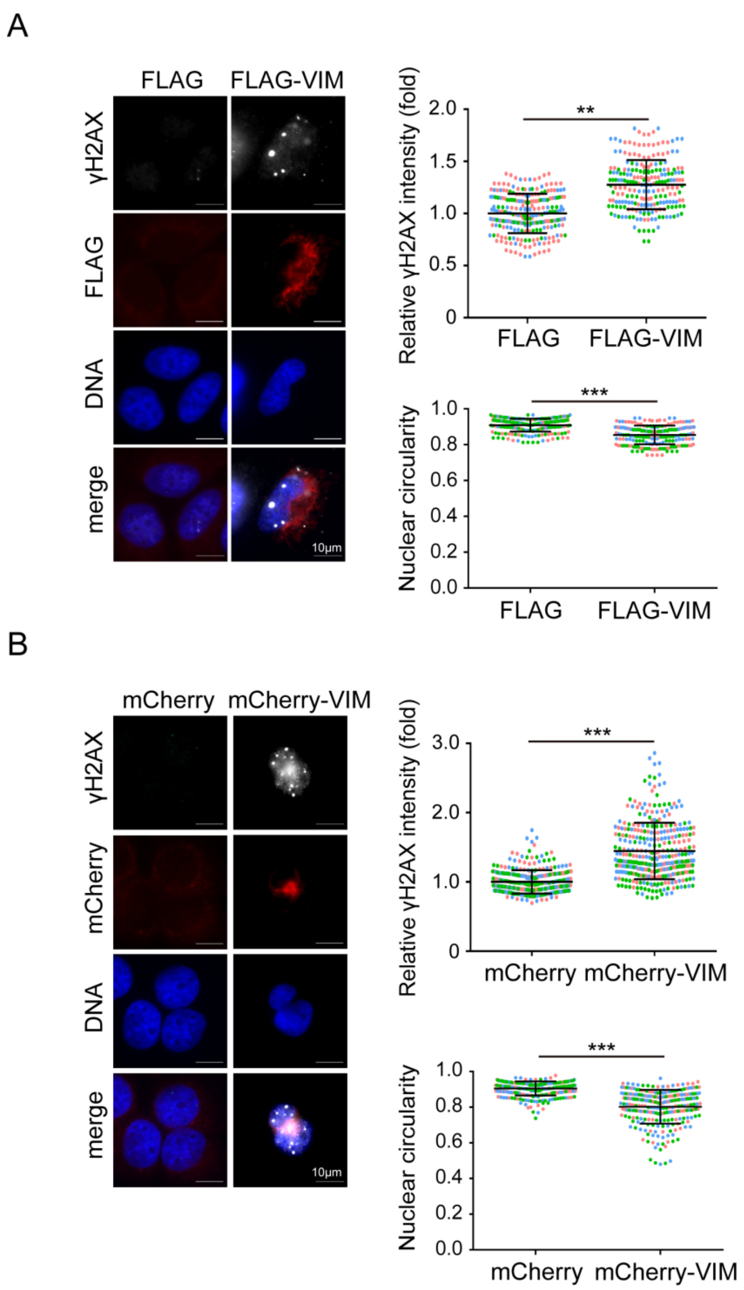

Figure S10. DNA damage is increased in the dysmorphic nucleus induced by ectopically expressed vimentin in MCF7 cells. (**A**) MCF7 cells were transiently transfected with the plasmid encoding FLAG-vimentin or the control vector for 24 h. The cells were stained for γH2AX (white), FLAG-vimentin (red), and DNA (blue). Scale bars, 10 µm. The fluorescence intensity of γH2AX in the transfection-positive cells was measured ($n \geq 123$) and expressed as –fold relative to the control. The nuclear circularity of the cells was determined ($n \geq 232$). (**B**) MCF7 cells were transiently transfected with the plasmid encoding mCherry-vimentin or the control vector for 24 h.The cells were stained for γH2AX (white), mCherry-vimentin (red), and DNA (blue). Scale bars, 10 µm. The fluorescence intensity of γH2AX in the transfection-positive cells was measured ($n \geq 324$) and expressed as –fold relative to the control. The nuclear circularity of the cells was determined ($n \geq 324$). Values (means ± SD) were from three independent experiments. **P<0.01, ***P<0.001.

A

MDA-MB-231 VIM$^{+/+}$ vs VIM$^{-/-}$ (RNA sequencing)			
No significance		significance	P value
DCLRE1A	PMS2P4	XRCC5	0.000721206
DCLRE1C	PMS2P5	DCLRE1B	0.006760697
ERCC1	RAD23A		
ERCC2	RAD23B		
ERCC4	RAD50		
ERCC5	RAD52		
ERCC6L	SFR1		
ERCC6L2	SPIDR		
ERCC8	SWI5		
MCM8	TONSL		
MCM9	TREX2		
MMS22L	XRCC1		
MRE11A	XRCC2		
PMS1	XRCC3		
PMS2	XRCC4		
PMS2P10	XRCC6		
PMS2P2	XRCC6P2		
PMS2P3			

B

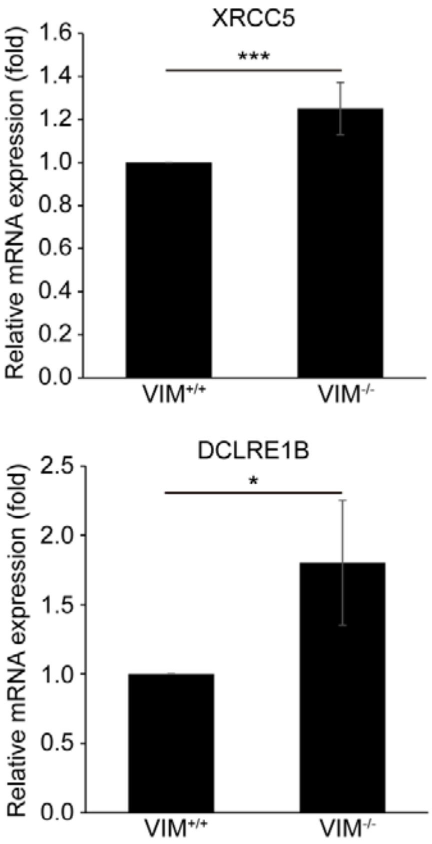

Figure S11. Vimentin gene knockout increases the expression of the DNA repair protein gene XRCC5 and DCLRE1B in MDA-MB-231 cells. (**A**) RNA-sequencing analysis was performed for MDA-MB-231 VIM$^{+/+}$ and VIM$^{-/-}$ cells. The transcripts of DNA repair genes were compared between VIM$^{+/+}$ and VIM$^{-/-}$ cells, among which XRCC5 and DCLRE1B were found to be statistically significant. (**B**) The mRNA levels of XRCC5 and DCLRE1B from MDA-MB-231 VIM$^{+/+}$ and VIM$^{-/-}$ cells were measured by quantitative real-time PCR and expressed as -fold relative to the MDA-MB-231 VIM$^{+/+}$ cells. Values (mean ± SD) were from three independent experiments. *$P < 0.05$ and ***$P < 0.001$.

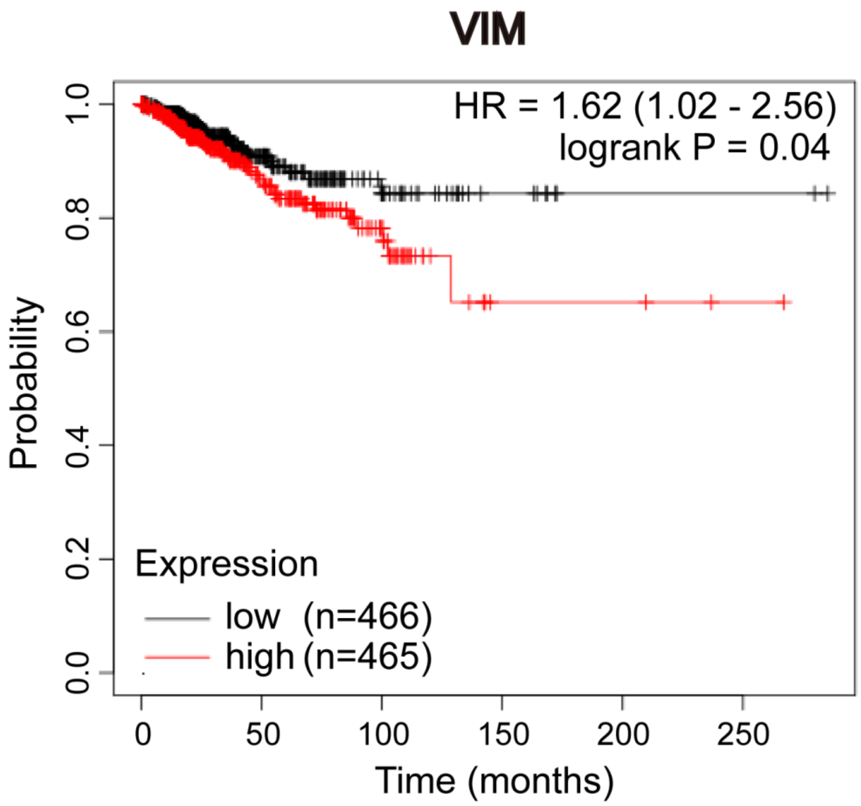

Figure S12. High expression of vimentin is correlated with a poor recurrence-free survival in breast cancer patients. The association between the expression level of vimentin and recurrence-free survival of the patients with breast cancer (stage 1-4) was analyzed using the online Kaplan-Meier plotter (https://kmplot.com/analysis).
